# Supplementary material for: Directed evolution of colE1 plasmid replication compatibility: a fast tractable tunable model for investigating biological orthogonality
Source: Nucleic Acids Res. 2022 Aug 26;50(16):9568–79. doi: 10.1093/nar/gkac682 (PMC9458437; doi:10.1093/nar/gkac682)
Supplement: gkac682_Supplemental_File [file gkac682_supplemental_file.pdf]

Title: Directed evolution of colE1 plasmid replication compatibility: a fast tractable tunable model for investigating biological orthogonality.

Authors:

Santiago Chaillou[*,1], Pinelopi-Eleftheria Stamou[*,2], Leticia Torres[2], Ana B. Riesco[2], Warren Hazelton[2], Vitor B. Pinheiro[1,#].

[1] KU Leuven, Department of Pharmaceutical and Pharmacological Sciences, Rega Institute for Medical Research, Herestraat, 49, 3000, Belgium

[2] University College London, Institute of Structural and Molecular Biology, University College London, Gower Street, London, WC1E 6BT, UK

* contributed equally to the publication.

# corresponding author: v.pinheiro@kuleuven.be

Supplementary information:

Supplementary Table 1: Primers used in this work 3

Supplementary Table 2: Number of events post single-cell gating used in the analysis of plasmid populations. 3

Supplementary Figure 1: Viable colE1 origins identified by NGS and screening. 4

Supplementary Table 3: Analysis by next generation sequencing of recovered viable origins. 4

Supplementary Figure 2: Compatibility selection in liquid culture. 5

Supplementary Figure 3: NGS analysis after large-scale selection for plasmid compatibility. 5

Supplementary Table 4: Analysis by next generation sequencing of recovered compatible origins. 6

Supplementary Figure 4: High-throughput screening assay for the selection of colE1-compatible origins of replication. 6

Supplementary Figure 5: Characterisation of selected colE1 origin variants for their compatibility with colE1. 7

Supplementary Figure 6: Impact of culture medium on plasmid compatibility and characterization of cross-compatibility. 9

Supplementary Figure 7: Sequence analysis of engineered colE1 origins and quantification of plasmid copy number per cell. 10

Supplementary Figure 8: Pairwise compatibility between origins in plasmids expressing fluorescent proteins. 11

Supplementary Figure 9: Plasmid intercompatibility assays. 12

Supplementary notes: 13

Appendix 1: Asymmetric plasmid compatibility simulated as a Lotka-Volterra system (Jupyter notebook running Julia 1.7.2) 14

Primer name	Primer sequence
**Construct and library assembly**	
ES VCTR FW	AAAGGTCTCAAGTGTAGCCGTAGTTAGGC
ES VCTR RV	AAAGGTCTCACAAGCAGCAGATTACGCG
ES ULTR RNAI FW	GGGGGTCTCACACTTAGAAG
ES ULTR REV	ACACACAGGTCTCACTTGC
ES COLEI ins GIBS FW	CTACGCATGGCTCAAAACACCCCTTGT
ES COLEI ins GIBS RV	TTTTTCCATAGGCTCCGCC
ES 002 FW	AAAGGTCTCACGTTAAAGGAAGCTGAGTTGGCT
ES 002 RV	AAAGGTCTCATAGAGGGGAATTGTTATCCGC
ES 003 FW	AAAGGTCTCATCTAGGGCTAACAGGAGGAATTAAC
ES 003 RV	AAAGGTCTCAAACGCATCCGCCAAAACAGC
ES BAD GFP FW	AAAGGTCTCACCCGTTTTTTGGGCTAAC
ES BAD GFP RV	AAAGGTCTCAGCTTCGCTTCTGCGTTCTGAT
ES PET GFP FW	AAAGGTCTCAAAGCCCGAAAGGAA
ES PET GFP RV	AAAGGTCTCACGGGAATTGTTATCCGCT
ES sfGFP plasm ampl FW	GGCGGAGCCTATGGAAAAA
ES sfGFP plasm ampl RV	GGGGTGTTTTGAGCCATGCGTAGAGGATCTGCTCA
SC_pBAD_dOri_FW	AAACGTCTCACTTGCATGTGTCAGAGGTTTTCAC
SC_pBAD_dOri_RV	AAACGTCTCATCACTCAGTGGAACGAAAACTCAC
SC_pBAD_dOri_dATB_RV	AAACGTCTCATCACTGTAGAAACGCAAAAAGGCC
SC_pET_addOri_addATB_FW	AAAGGTCTCAGTGACGTTTACAATTTCAGGTGGC
SC_pET_addOri_addATB_RV	AAAGGTCTCACAAGATCAGCTCACTCAAAGGC
SC_pWH_addOri_addATB_FW	AAAGGTCTCAGTGATTCCGTGATGGTAACTTCAC
SC_pWH_addOri_FW	AAAGGTCTCAGTGAGCAAGGATCTTCTTGAGATCC
SC_pWH_addOri_addATB_RV	AAAGGTCTCACAAGAATCATCTGGCCATTCGATG
AR_pWHalpha_Fw	TATGGAAAAACGCCAGCAACG
AR_pWHalpha_Rv	AAGATCCTTGCACTCGAGTTGATCG
VP023F	TTTGGTCTCA AAGTTGCACTCGAGTTGATCGGGC
VP023R	TTTGGTCTCA TTCCGCCTTTTTACGGTTCCTGGCC
WH81	TCCTCGAGGCTTGGATTCTC
WH82	TGCACTCGAGTTGATCGGG
WH83	TGCCCGATCAACTCGAGTGCAAGGATCTTCTTGAGATCC
WH84	AACGAGACATCATTTTTTGCCCTCGTTATCTAG
WH85	AGGGCAAAAAATGATGTCTCGTTTAGATAAAAG
WH86	AGAATCCAAGCCTCGAGGAAGATCCTTTGATCTTTTCTAC
WH87	TCCCTATCAGTGATAGAGAACCTCTAGAAATAATTTTGTTTAAC
WH88	ATCAATGATAGAGTGTCAACATTTCGCGGATCGAG
TetA	GTTGACACTCTATCATTGATAGAGTTATTTTACCACTCCCTATCAGTGATAGAGAA
TetAR	TTCTCTATCACTGATAGGGAGTGGTAAAATAACTCTATCAATGATAGAGTGTCAAC
Sequencing	
ES seq-ing 001	TCACTCAAAGGCGGTAA
ES seq-ing 002	TGTCGGGTCATGTGAGCAA
ES seq-ing 002 FW	ATGGCTCATAACACCCCTTGT
NGS_Forward primer	TTCTGCGCGTAATCTGCTGC
NGS_Reverse primer	GGCCTAACTACGGCTACACTAG
ES DEEP SEQ PET ini FW	AATGATACGGCGACCACCGAGATCTACACTCTTTCCCTACACGACGCTCTTCCGATCTNNNT<br>GACCATTCTGCGCGTAATCTGCTGC
ES DEEP SEQ PET vai FW	AATGATACGGCGACCACCGAGATCTACACTCTTTCCCTACACGACGCTCTTCCGATCTNNN<br>ACAGTGTTCTGCGCGTAATCTGCTGC
ES DEEP SEQ PET RV	CAAGCAGAAGACGGCATACGAGATCGGTCTCGGCATTCCTGCTGAACCGCTCTTCCGATCTG<br>GCCTAACTACGGCTACACTAG

Digital PCR	
SC_dPCR_Chl_FW	AATAAAGGCCGGATAAAACTTG
SC_dPCR_Chl_RV	CTGGATATACCACCGTTGATAT
SC_dPCR_Chl_probe	/56-FAM/AATATCCAG/ZEN/CTGAACGGTCTGG/3IABKFQ/
SC_dPCR_ter_FW	AATAACATTCATTGGGTTGGTC
SC_dPCR_ter_RV	GCATGGTTAATCACGATGTAAT
SC_dPCR_ter_probe	/5HEX/AATAGCTAC/ZEN/CTCATCCGCGAAG/3IABKFQ/

Supplementary Table 1: Primers used in this work. All primers are shown in 5'→3' orientation. Chemical modifications for the primers used in digital PCR were as follows: /56-FAM/ - fluorescein; /ZEN/ - ZEN^{TM} quencher; /3IABKFQ/ - Iowa Black® FQ; /5HEX/ - Hexachlorofluorescein.

Plasmid combination	# events
**Intercompatibility experiments**	
**D4_1 (all)**	2754
**D4_1 (CM only)**	43973
**D4_1 (no ATB)**	9029
**G6 (all)**	6736
**G6 (CM only)**	10225
**G6 (no ATB)**	3597
**G4 (all)**	2417
**G4 (CM only)**	9626
**G4 (no ATB)**	3046
**Pairwise_intercompatibility experiments**	
**D4_2 + colE1 (CM)**	8804
**D4_2 + colE1 (no ATB)**	6896
**D4_1 + colE1 (no ATB)**	1520
**colE1 + G4 (no ATB)**	8195
**colE1 + G6 (no ATB)**	5304
**D4_1 + D4_2 (CM only)**	1903
**D4_1 + D4_2 (no ATB)**	6851
**D4_2 + G6 (CM only)**	2625
**D4_2 + G6 (no ATB)**	9647
**D4_2 + G4 (CM only)**	1399
**D4_2 + G4 (no ATB)**	2428

Supplementary Table 2: Number of events post single-cell gating used in the analysis of plasmid populations. Naming of the experiments refers to the origins from each plasmid as described in the main text. Compatibility experiments carried out in the presence of kanamycin, chloramphenicol and ampicillin are shown as (all). Where only chloramphenicol was used in the experiment, samples are shown as (CM only). Experiments carried out in the absence of any antibiotic are shown as (no ATB).

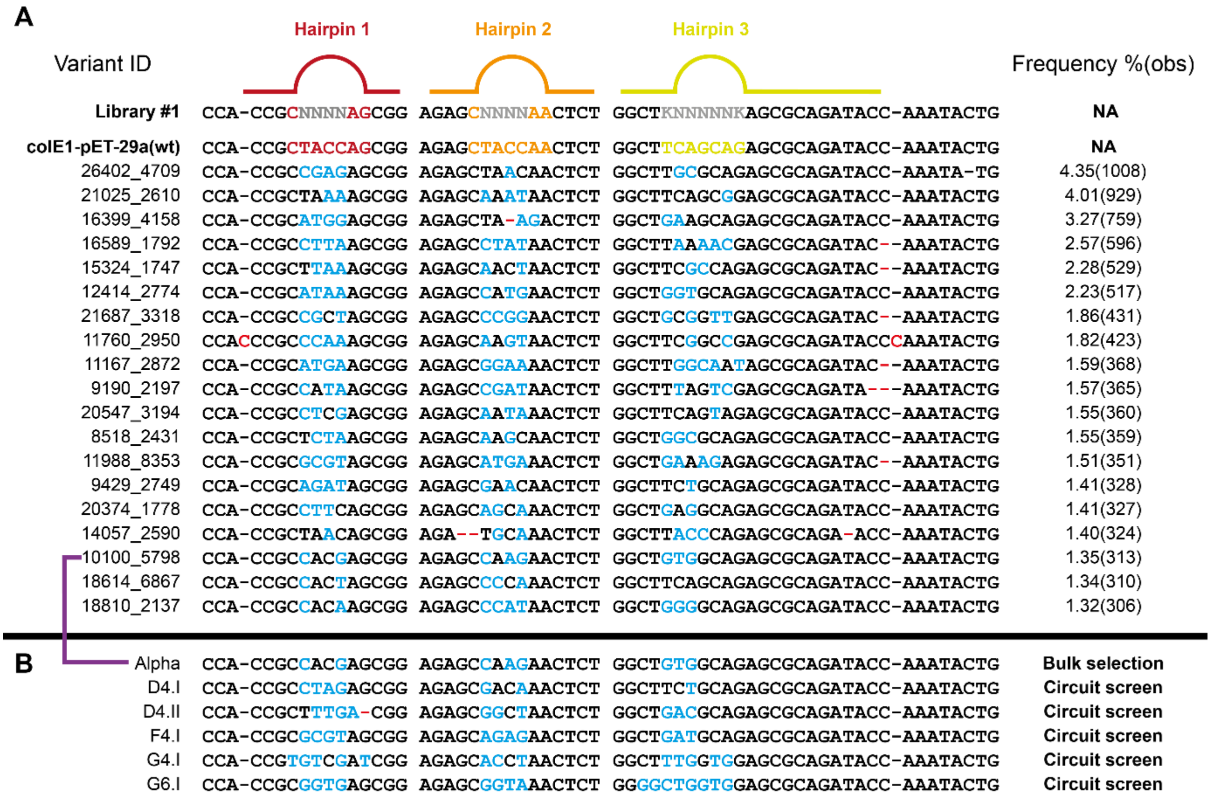

Supplementary Figure 1: Viable colE1 origins identified by NGS and screening. A. NGS analysis of viable colE1 origins isolated from transformation of library #1. Mutations away from the wild-type sequence introduced by the library are shown in blue, mutations arising from selection are shown in red. Frequency of isolated origins is shown with the individual number of observations in brackets. The ID (automatically generated in sequencing) of one of the unique sequences is picked (arbitrarily) to name the group. NA – not applicable. **B.** Engineered colE1 origins described in this work.

Pipeline step	Sequences output
Total read number	31144
Quality trimming	31144 (100%)
Filtering by 5' sequence	29916 (96%)
Filtering by 5' sequence #2	29151 (94%)
Filtering by 3' sequence	27175 (87%)
Filtering by 3' sequence #2	23183* (74%)
Unique sequences	1903

Supplementary Table 3: Analysis by next generation sequencing of recovered viable origins. Total read number obtained and the impact of the analysis pipeline are shown. *Number of sequences used in downstream analysis.

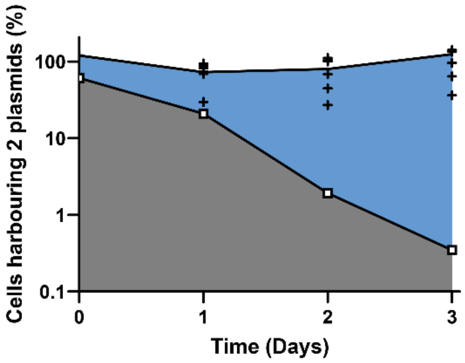

Supplementary Figure 2: Compatibility selection in liquid culture. *E. coli* harbouring pSB1C3 (colE1 origin) and transformed with pET29 containing its wild-type (colE1; white squares) or a library of viable origins (black crosses) were serially passaged, with samples plated in the absence of antibiotics (to determine total CFU) or in the presence of both antibiotics (to determine CFU still harbouring both plasmids). As expected, under the growth conditions, the wild type colE1 origin is rapidly lost from the population.

A

Variant ID	Hairpin 1	Hairpin 2	Hairpin 3	Frequency %(obs)
**colE1_pET-29a(wt)**	CCACCGCTACCAGCGG	AGAGCTACCAACTCT	GGCTTCAGCAGAGCGCAGATACCAAATACTG	**NA**
19921_1932	CCACCGCTACCAGCGG	AGAGCTACCAACTCT	GGCTTCAGCAGAGCGCAGATACCAAATACTG	(13432)
24147_3005 (alpha)	CCACCGCCACGAGCGG	AGAGCCAAGAACTCT	GGCTGTGGCAGAGCGCAGATACCAAATACTG	11.22(1433)
16596_2335	CCACCGCACCGAGCGG	AGAGCCGCCAACTCT	GGTTGACTAAGAGCGCAGATACCAAATACTG	4.01(512)
15490_2347	CCACCGCCATAAGCGG	AGAGCCGCCAACTCT	GGCTGGCAGAGAGCGCAGATACCAAATACTG	3.63(464)
23482_1919	CCACCGCAGAGAGCGG	AGAGCCCAAAACTCT	GGCTTCGGCAGAGCGCAGATACCAAATACTG	3.58(457)
11945_7277	CCACCGCACCTAGCGG	AGAGCCATCAACTCT	GGCTGACGCAGAGCGCAGATACCAAATACTG	3.52(450)
21796_3950	CCACCGCACATAGCGG	AGAGCTTTCAACTCT	GGCTTGAGCAGAGCGCAGATACCAAATACTG	3.21(410)
6435_3326	CCACCGCCACTAGCGG	AGAGCCCCAAACTCT	GGCTTCAGCAGAGCGCAGATACCAAATACTG	2.72(347)
13190_7993	CCACCGCAGAGAGCGG	AGAGCCCTCAACTCT	GGCTGTTGCAGAGCGCAGATACCAAATACTG	2.51(321)
13128_4999	CCACCGCCCACAGCGG	AGAGCCAGCAACTCT	GGCTGAGTAAGAGCGCAGA-ACCAAATACTG	2.47(315)
13613_5267	CCACCGCCACGAGCGG	AGAGCTACAAACTCT	GGCTTCGACAGAGCGCAGA-ACCAAATACTG	2.43(310)
16898_3487	CCACCGCCATGAGCGG	AGAGCCCGCAACTCT	GGCTTGACCTGAGCGCAGATA-CAAATACTG	1.97(252)
25074_2902	CCACCGCAGACAGCGG	AGAGCCTCTAACTCT	GGCTGACGCTGAGCGCAGA-ACCAAATACTG	1.96(251)
21215_6358	CCACCGCACCAAGCGG	AGAGCGCTTAACTCT	GGCTTACGCAGAGCGCAGATACCAAATACTG	1.96(250)
12850_3238	CCACCGCCCTAAGCGG	--AGCCATTAACTCT	GGCTTATGCAGAGCGCAGATACCAAATACTG	1.78(227)
25300_8619	CCACCGCAGATAGCGG	AGAGCCAACAACTCT	GGCTTCCGCAGAGCGCAGATACCAAATACTG	1.76(225)
14580_2678	CCACCGCCACCAGCGG	AGAGCTATTAACTCT	GGCTTCGGCAGAGCGCAGATACCAAATACTG	1.73(221)

Supplementary Figure 3: NGS analysis after large-scale selection for plasmid compatibility. NGS analysis of colE1 origins isolated after selection of viable colE1 variants co-transformed with wild-type colE1. Mutations away from the wild-type sequence introduced by the library are shown in blue, mutations arising from selection are shown in red. Frequency of isolated origins is shown with the individual number of observations in brackets. Wild-type colE1 sequences were identified in the experiment (a limitation of the approach used to prepare plasmid DNA for NGS) and are excluded from the analysis – the number of observations is still given. The ID (automatically generated in sequencing) of one of the unique sequences is picked (arbitrarily) to name the group. NA – not applicable.

Pipeline step	Sequences output
**Total read number**	28438
**Quality trimming**	28438 (100%)
**Filtering by 5' sequence**	27876 (98%)
**Filtering by 5' sequence #2**	27708 (97%)
**Filtering by 3' sequence**	26777 (94%)
**Filtering by 3' sequence #2**	26206* (92%)
**Unique sequences**	1185

Supplementary Table 4: Analysis by next generation sequencing of recovered compatible origins. Total read number obtained and the impact of the analysis pipeline are shown. *Number of sequences used in downstream analysis.

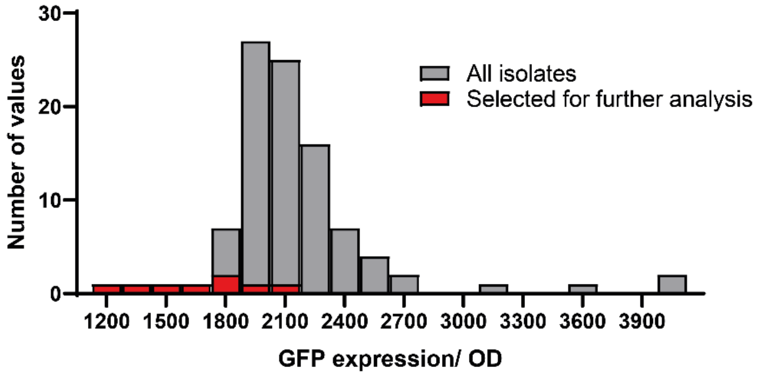

Supplementary Figure 4: High-throughput screening assay for the selection of colE1-compatible origins of replication. Histogram representation of the data shown in Figure 3C, highlighting the fluorescence values of the variants selected for further study.

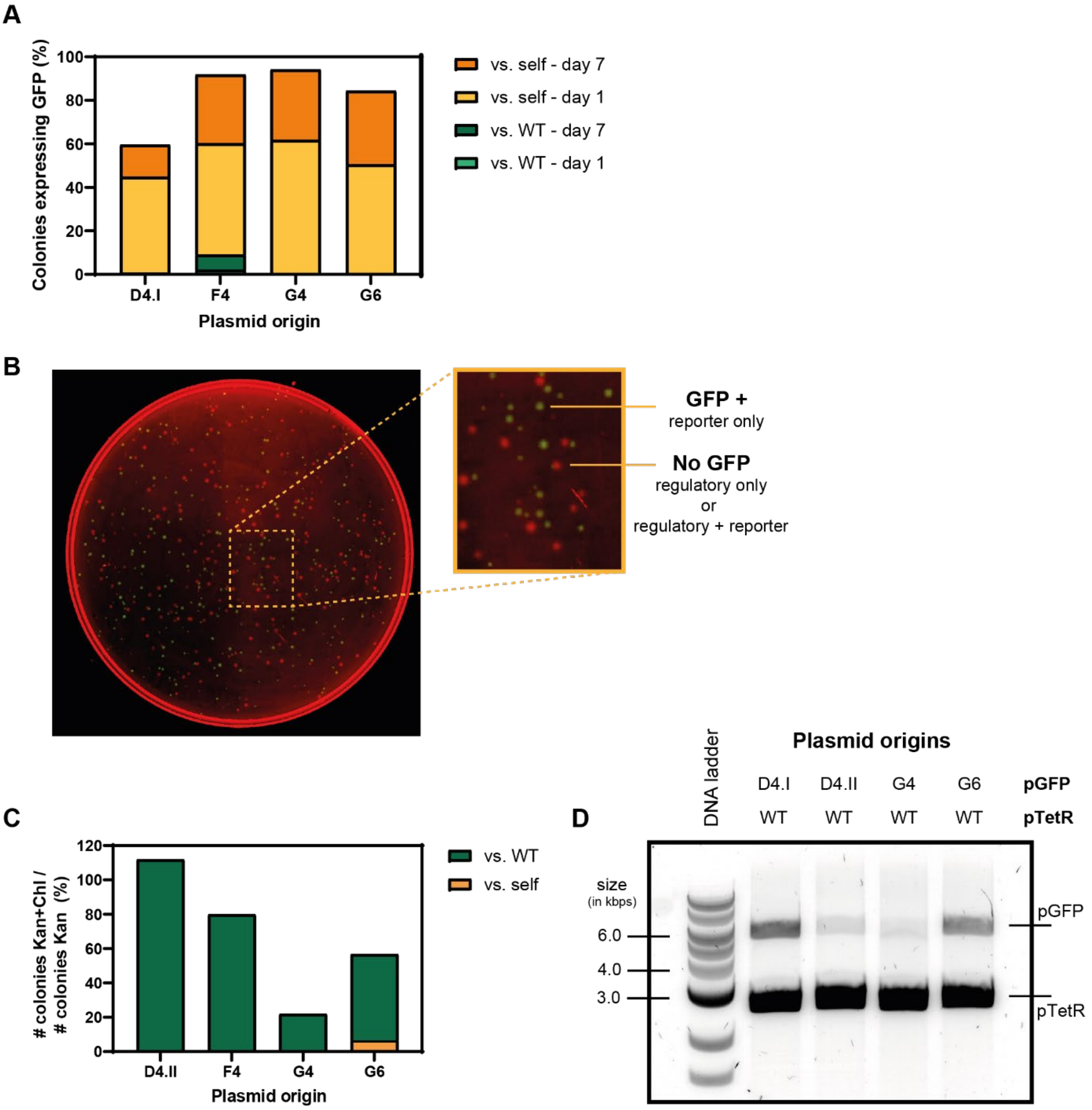

Supplementary Figure 5: Characterisation of selected colE1 origin variants for their compatibility with colE1. A. Serial cultures of *E. coli* cells co-transformed with reporter (harbouring one of D4.I, F4, G4, G6 and wild-type origins of replication) and regulatory (harbouring D4.I, F4, G4 or G6 origins) were used to test the compatibility of the selected variants against wild-type and to confirm their self-incompatibility. The percentage of cells expressing GFP was calculated by diluting a culture aliquot (after 1 or 7 days of passaging) and plating in LB agar supplemented with chloramphenicol (to retain reporter plasmid). Bar graphs are overlaid with compatibility to wild-type shown in green (light or dark depending on passage number) and self-incompatibility shown in orange (light or dark depending on passage number). **B.** Example of transformation plate used to calculate values in **A.** Here, D4.I was used in both regulatory and reporter plasmids and the results show the distribution of plasmids after 7 days of passaging. Fluorescent images from GFP (green) and control channels (red) are overlaid and CFU counted. **C.** Complementary experiment where after passaging in the absence of antibiotic selection, cultures are plated in media supplemented with kanamycin (regulatory plasmid antibiotic marker) or with both antibiotics to monitor plasmid loss. Bar graphs are overlaid with compatibility to wild-type shown in green and self-incompatibility shown in orange. **D.** Plasmids isolated after coc-

culture experiments, showing that both plasmids are retained throughout the experiment. Notably, it is possible to see the variation of copy number between the origins described and how D4.II has a lower copy number than G6 (in contrast to Figure 4C) when in the presence of wild-type origin. Evolved origins are present in the reporter construct (pGFP) while wild-type origins were used in the regulatory plasmid (pTetR). Transformation of isolated reporter constructs confirm that most are still able to express GFP (data not shown). Sequencing of isolated reporter constructs from this experiment confirm that promoter, RBS and GFP gene did not acquire any mutations within the experimental time frame (data not shown). While not shown, the data are available in the online Github repository.

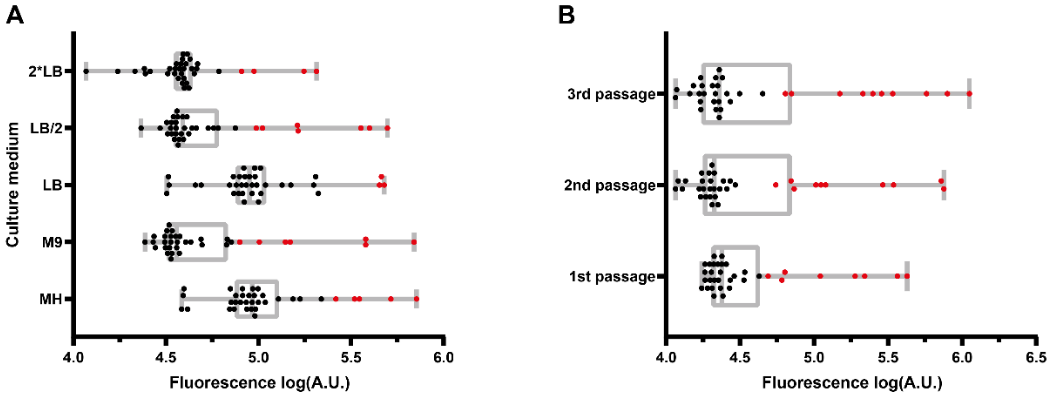

C

1st dilution							
M9		pGFP(TetA)					
		WT	D4 I	G6	D4 II	F4	G4
pTetR	WT		28838	27170	24209	23776	23285
	D4 I	19704	34078	25503	18343	20212	20976
	G6	23319	220142	365037	22609	20490	22387
	D4 II	21133	42589	21106	60662	18349	18293
	F4	19862	26849	21184	19061	63579	17417
	G4	33635	110283	425005	23830	48928	189417

pGFP(TetA) positive control	387180
pTetR negative control	19953

3rd dilution							
M9		pGFP(TetA)					
		WT	D4 I	G6	D4 II	F4	G4
pTetR	WT		21699	21105	22897	15320	16763
	D4 I	20591	574962	17190	17078	17176	18047
	G6	27180	213569	796289	22905	14470	21593
	D4 II	22785	64041	11803	339829	21765	23394
	F4	22291	45029	11608	24051	249330	17521
	G4	25794	70443	1119908	31474	149623	286265

pGFP(TetA) positive control	738296
pTetR negative control	22328

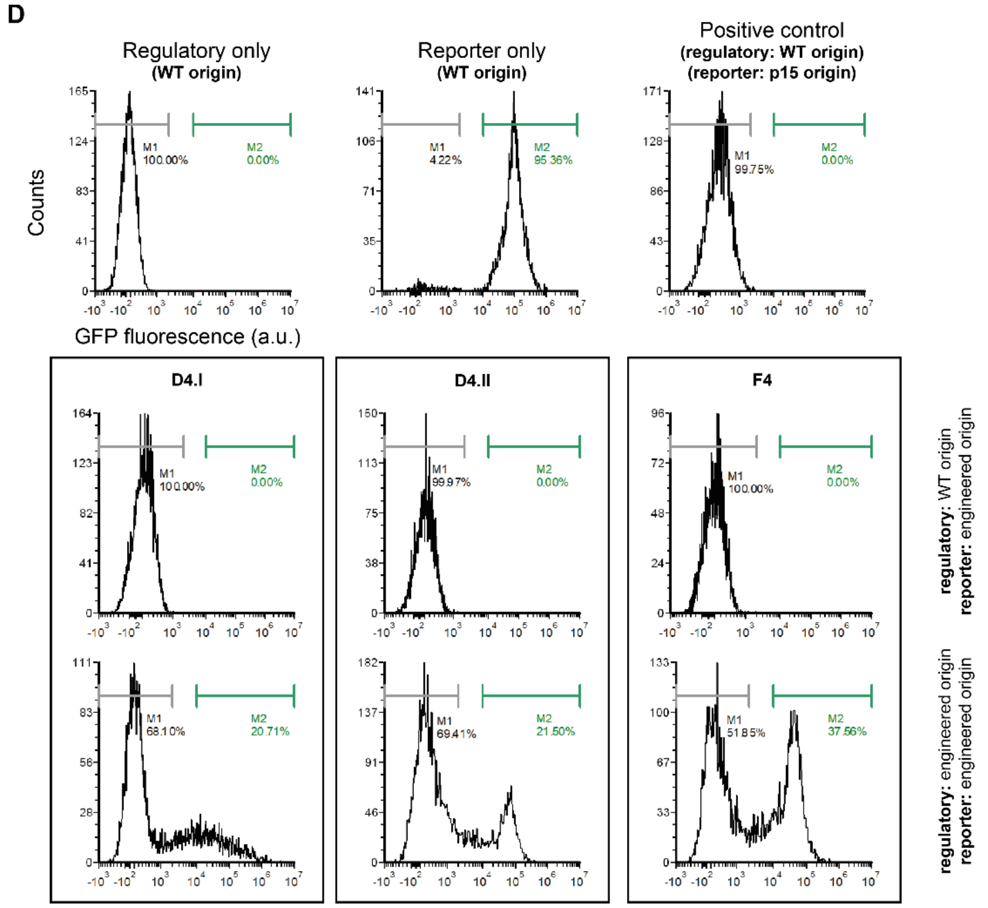

Supplementary Figure 6: Impact of culture medium on plasmid compatibility and characterization of cross-compatibility. A. Box plot showing the distribution of normalized fluorescence for cross-compatibility assays carried out in different culture media (single passage). Outliers (2% cut-off in ROUT analysis), that is significantly expressing GFP, are shown in red. **B.** Box plot showing the impact of passaging in cross-compatibility assays in M9 media. Outliers (2% cut-off in ROUT analysis), that is

significantly expressing GFP, are shown in red. **C.** Cross-compatibility results (normalized fluorescence values) obtained in M9 after one or two passages. Outliers identified in **B.** are shown in green. The third passage is shown in Figure 4A. **D.** Flow cytometry analysis of cross-compatibility assays showing controls and selected replication origins, their compatibility to wild-type colE1 origins and their self-incompatibility. Markers show ranges used to quantify non-fluorescence (grey) and fluorescent (green) fractions of the populations. Each experiment included at least 6200 events post single-cell gating.

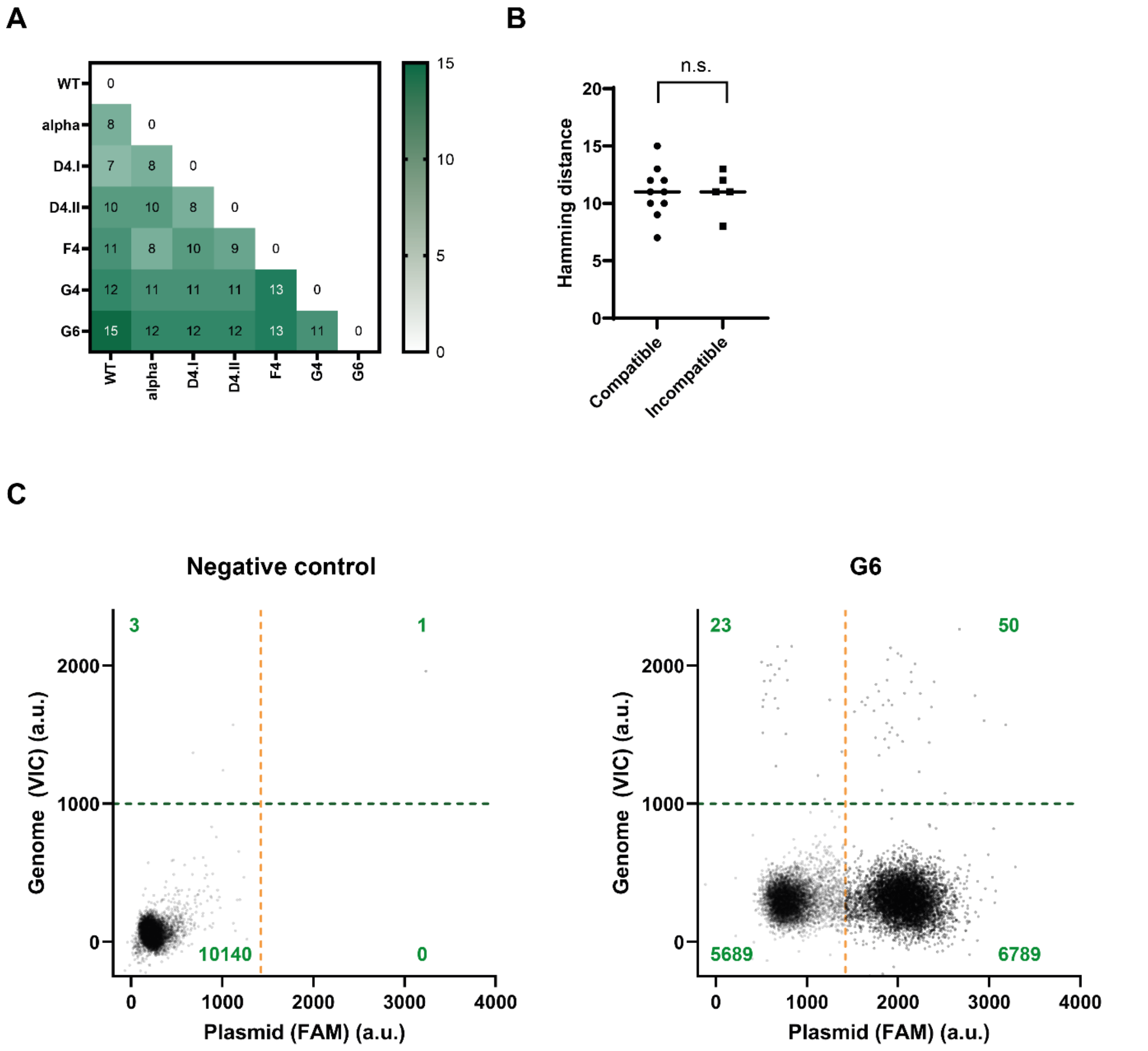

Supplementary Figure 7: Sequence analysis of engineered colE1 origins and quantification of plasmid copy number per cell. A. Hamming distance (number of substitutions between 2 sequences) between the engineered origins of replication. **B.** Hamming distance distribution between compatible and incompatible colE1 origins (using data presented in Figure 4A). A Kolmogorov-Smirnov test was used to compare the two Hamming distance distributions but no significant different was observed. **C.** Examples of digital PCR results to show negative control (no template) and results obtained for G6

engineered origin. The quadrants are determined automatically by the analysis program and the number of observations in each quadrant are shown in green.

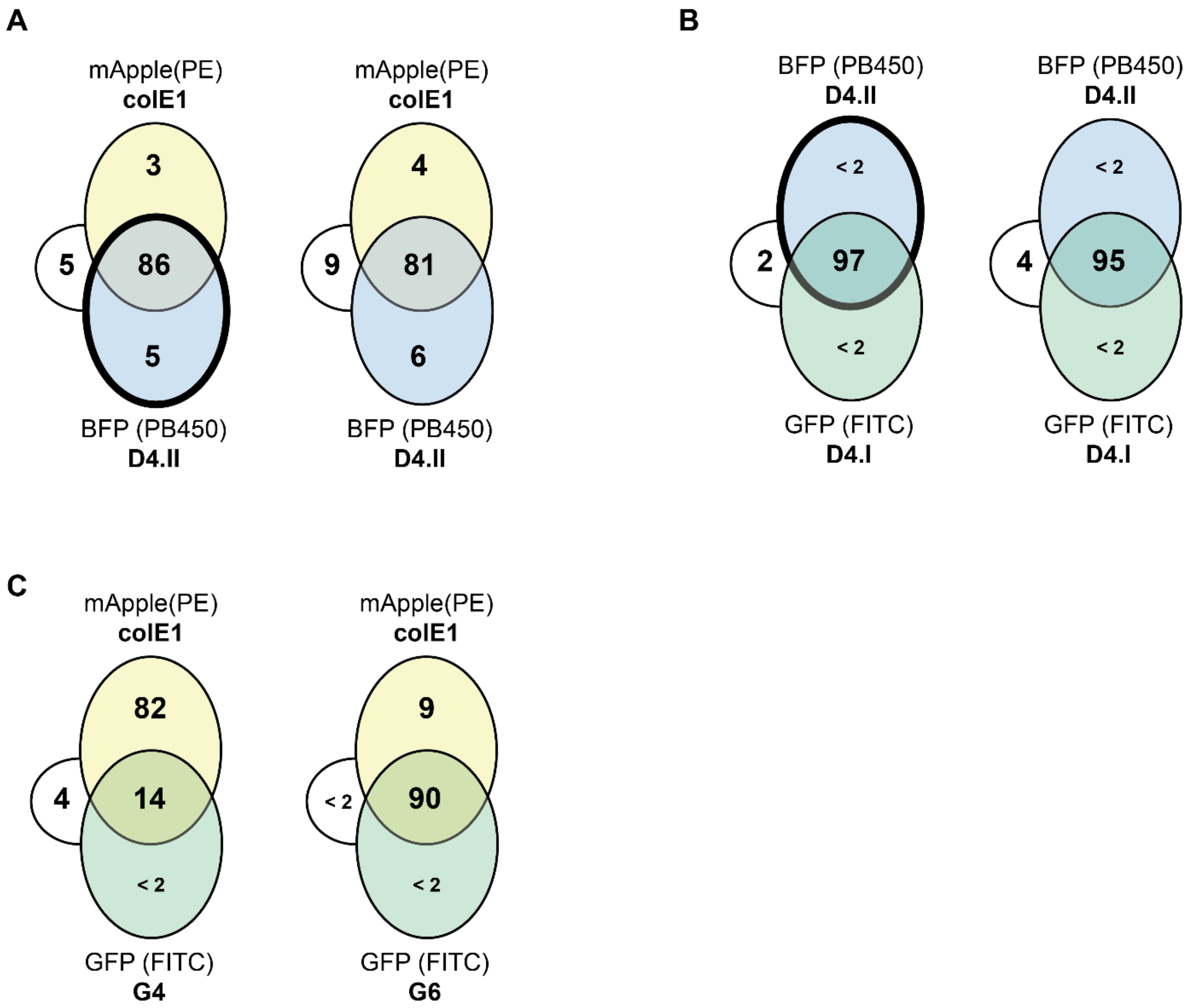

Supplementary Figure 8: Pairwise compatibility between origins in plasmids expressing fluorescent proteins. Summary of flow cytometry analysis of cultures post-serial passaging in M9 used to investigate plasmid retention and plasmid compatibility. Origins and fluorescent protein encoded are shown around the edges of the Venn diagram: D4.II origin in mTagBFP2-pBAD (blue), colE1 origin in mApple-pBAD (yellow) and other origins in GFP-pBAD (green). Thick borders show experiments where chloramphenicol was used to ensure D4.II plasmid retention. **A.** D4.II and colE1 origins. **B.** D4.I and D4.II origins. Both show that chloramphenicol selection has little impact on the retention of the plasmids. **C.** colE1 origins and G4 or G6. Under the culture conditions used for this experiment, the G4 origin is lost from the population (in alignment with what was seen in SI Fig 5C, but different from what was observed in the high-throughput assay (Figure 4A). These experiments were also used as controls for the 3-way intercompatibility assays.

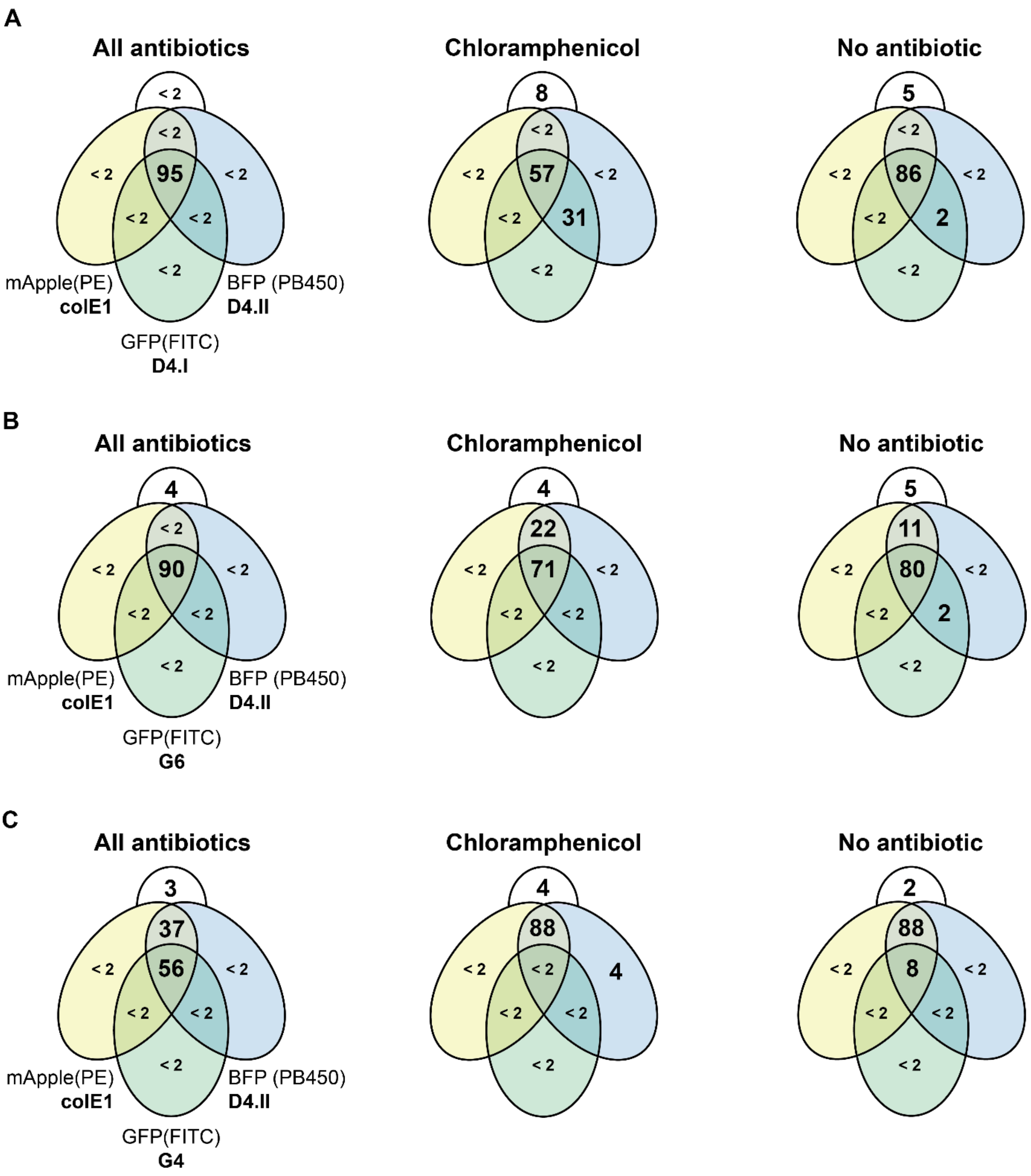

Supplementary Figure 9: Plasmid intercompatibility assays. Summary of flow cytometry analysis of cultures post-serial passaging in M9 used to investigate plasmid retention and plasmid compatibility. Cells co-transformed with three plasmids harbouring different plasmid origin combinations were serially passaged in M9 before being analysed by flow cytometry to determine which plasmids had been retained in culture. Plasmid origins and fluorescent proteins are shown for each combination around the Venn diagram. BFP is shown in blue, mApple in yellow and GFP in green. Cultures were maintained with all antibiotics (ampicillin, chloramphenicol and kanamycin), or with only chloramphenicol, or without any added antibiotics. **A.** Origins D4.I, D4.II and wild-type colE1. Passaging of the culture in the presence of chloramphenicol results in significant wild-type colE1 loss. **B.** Origins G6, D4.II and colE1. Plasmid harbouring G6 origin is preferentially lost from culture but at slow rates, ensuring that most of the population retains all 3 plasmids. **C.** Origins G4, D4.II and wild-type colE1. In contrast to the pairwise assays, plasmids with the G4 origin were rapidly lost from the

population, even in the presence of all three antibiotics, suggesting that it may not as stable as other origins or that its low copy number puts it in a significant disadvantage during replication.

Supplementary notes:

Polymerase Chain Reaction. PCR was used to generate the biological constructs for this work. Unless stated otherwise, all reactions were carried out in 50 µL with the following reaction components: 1X Q5 reaction buffer, 0.5 µM of each primer, 200 µM dNTPs, 0.2 ng/µL of template, 0.02 U/µl Q5 enzyme (New England Biolabs), and deionized sterile water to complete the reaction volume. The reaction conditions typically consisted of an initial denaturation at 95°C for 30 seconds, followed by 30 – 32 cycles of 95°C for 20 seconds, 50 - 72°C for 30 seconds, 72°C for 30 seconds/kb of the target DNA product. All reactions included final 72°C extension for 5 minutes.

Raw data. All data and analyses generated in this project are publicly available at https://github.com/PinheiroLab/Engineered_colE1_origins. Sequences for the newly described colE1 origins have been deposited on GenBank under the following accession numbers: OL702929, OL702930, OL702931, OL702932, OL702933 and OL702934. Next generation sequencing data has been deposited on NCBI SRA under the following accession number: PRJNA783752.

Appendix 1: Asymmetric plasmid compatibility simulated as a Lotka-Volterra system (Jupyter notebook running Julia 1.7.2)

Asymmetric plasmid compatibility simulated as a Lotka-Volterra system

Vitor Pinheiro (v1.0) - 15.05.22

```
## Packages required for calculation and visualization
using DifferentialEquations
using Plots
```

Setting up a 2-population Lotka-Volterra (LV) system

```
function two_plasmid_competition!(du, u, p, t) ## 2-population generic LV system
    A, B = u
    a1, a2, a3, b1, b2, b3 = p
    du[1] = dA = a1*A - a2*A^2 + a3*A*B
    du[2] = dB = b1*B - b2*B^2 + b3*A*B
end
```

```
two_plasmid_competition! (generic function with 1 method)
```

```
## Parameters
a1 = 1  #= For simplicity we have placed a1 and b1 as 1. Consequently, time does not represent a convenient unit (e.g. minutes)=#
a2 = 1/100 ## a1/a2 is the carrying capacity of plasmid A
#= More accurately, the carrying capacity needs to be represented as 1/(x+y), where x refers to the carrying capacity
 without antibiotic selection and y is the increase in copy number driven by antibiotic selection =#
a3 = - 7/1000 ## impact of B on A
b1 = 1
b2 = 1/50 ## b1/b2 is the carrying capacity of plasmid B
b3 = - 17/1000 ## impact of A on B

## Initial conditions
#= Because experimentally cells are grown in the presence of both antibiotics, the initial conditions
should be the carrying capacity of each of the plasmids. Nevertheless, this type of LV system always converges
towards a single equilibrium point. Therefore most starting conditions will lead to the same long-term result =#
a0 = 1/a2
b0 = 1/b2

## Reformatting parameters for function
p = [a1, a2, a3, b1, b2, b3]
u0 = [a0, b0];

## Solution

#= While the LV system can be solved analytically, we provide here the num
```

```
erical solution to avoid having to introduce error
    checking for equilibrium positions that are not in the real positive s
pace for both populations. =#
tspan = (0.0, 100.0)  # gives the model 100 units of time to run
problem = ODEProblem(two_plasmid_competition!, u0, tspan, p)
solution = solve(problem, AutoVern7(Rodas5()), dt=0.1)

solution(100)  ## returns the two plasmid populations at t = 100

2-element Vector{Float64}:
 99.9999955110674
  2.621838197958631e-15

## Plots the two populations as a function of time
plot(solution, vars=(0,2), linewidth = 3, ylims = (0.0,max(a0,b0)), label
= "B(t)")
plot!(solution, vars=(0,1), linewidth = 3, label = "A(t)")
```

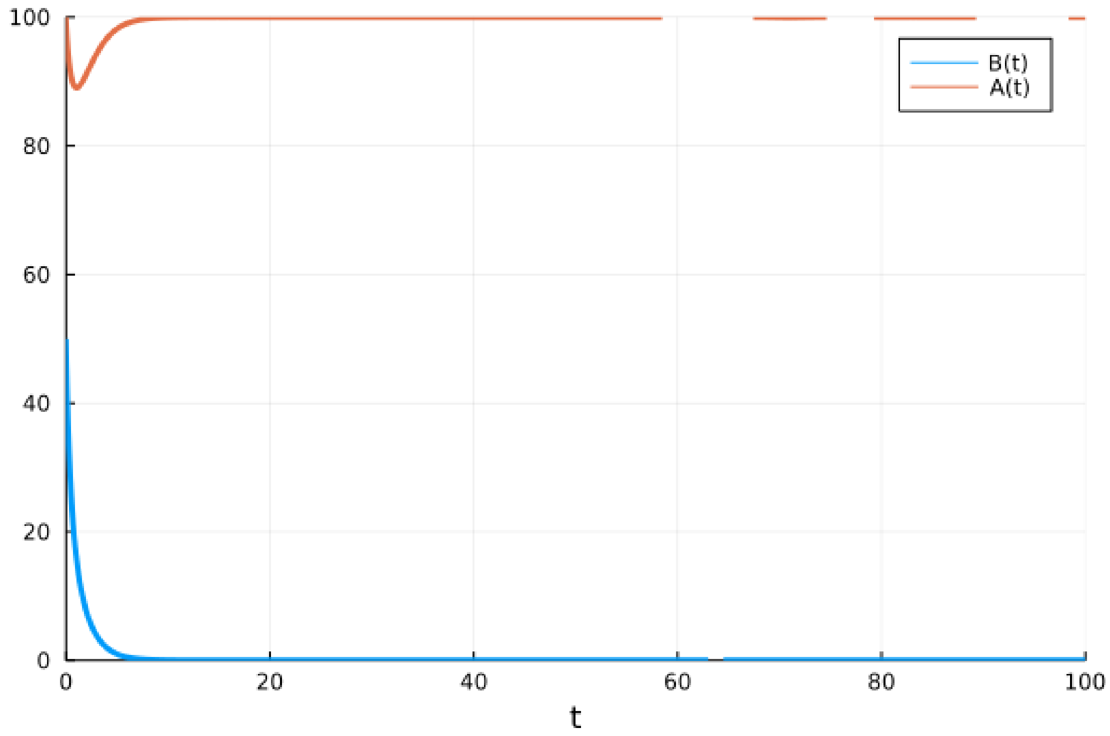

Analysing the parameter space of a 2-population LV system

```
## Solving the predicted interaction across a large window of interaction
parameters
a3_range = -0.02:0.0001:0  # sets up the range of the investigation
b3_range = -0.02:0.0001:0

results_A = zeros(length(a3_range), length(b3_range)) # prepares a matrix
for entering the results
results_B = zeros(length(a3_range), length(b3_range))

for i = 1:length(a3_range)
    for j = 1:length(b3_range)
        a3 = a3_range[i]
        b3 = b3_range[j]
```

```
        p = [a1, a2, a3, b1, b2, b3]
        u0 = [a0, b0];
        tspan = (0.0, 100.0)  # gives the model 100 units of time to run
        problem = ODEProblem(two_plasmid_competition!, u0, tspan, p)
        solution = solve(problem, AutoVern7(Rodas5()), dt=0.1)

        results_A[i,j] = solution(100)[1]
        results_B[i,j] = solution(100)[2]
    end
end


## Effect on population A
heatmap(a3_range, b3_range, results_A, c = :thermal, xlabel = "Effect of o
riA on oriB (b3)",
 ylabel = "Effect of oriB on oriA (a3)", title = "Plasmid A", clims = (0.0
,max(a0,b0)))
```

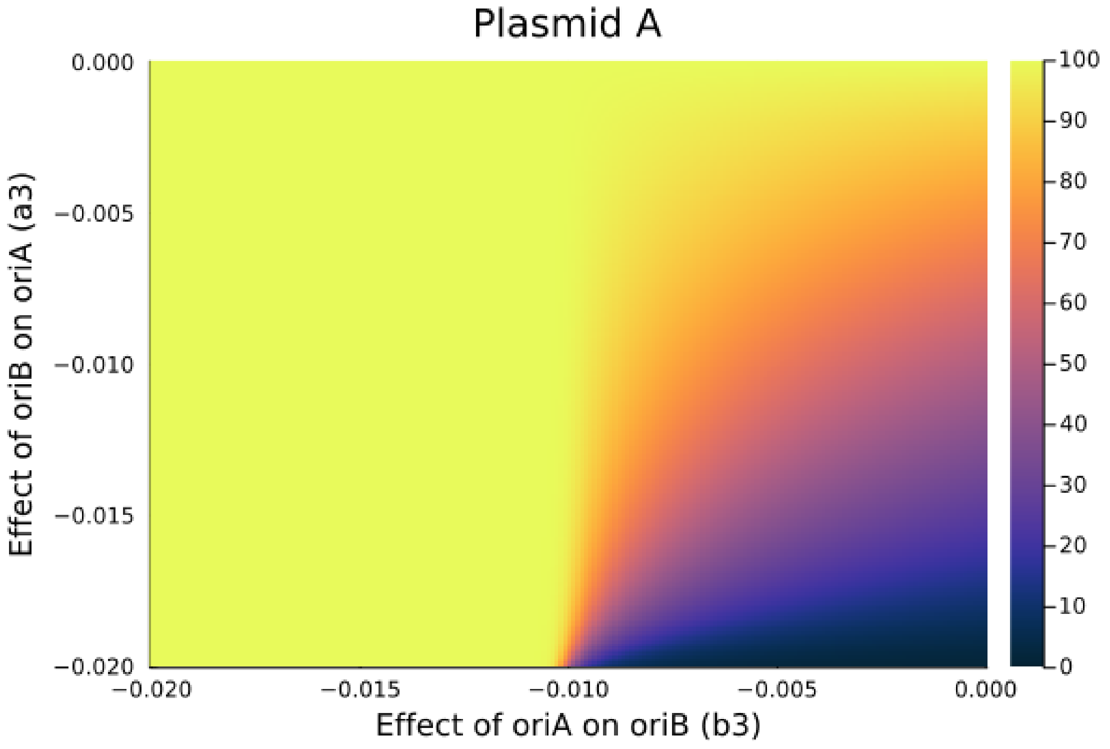


```
## Effect on population B
heatmap(a3_range, b3_range, results_B, c = :thermal, xlabel = "Effect of o
riA on oriB (b3)",
 ylabel = "Effect of oriB on oriA (a3)", title = "Plasmid B", clims = (0.0
,max(a0,b0)))
```

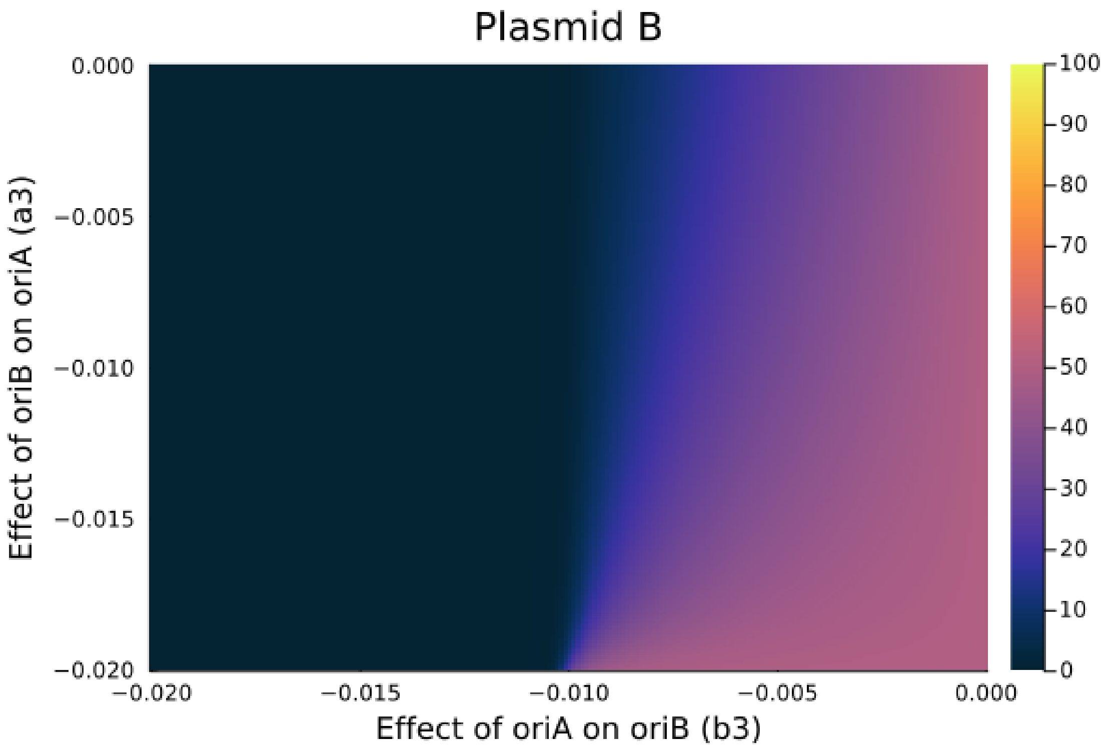


```
## Determining areas of co-existence
compatibility = zeros(length(a3_range), length(b3_range)) # prepares a mat
rix for entering the results

for n = 1: length(results_A)
    if results_A[n] <= 1 || results_B[n] <= 1
        #= Because the system is continuous, it tolerates very small numbe
rs which would have no real meaning
        in a discrete system. As such, we have used here a cut-off of 1, b
elow which the discrete nature of the real system
        would break down =#
        compatibility[n] = 0.0
    else
        compatibility[n] = 1.0
    end
end
heatmap(a3_range, b3_range, compatibility, c = :blues, colorbar=false, xla
bel = "Effect of oriA on oriB (b3)",
ylabel = "Effect of oriB on oriA (a3)", title = "Compatibility parameter s
pace")

# light blue = incompatible, dark blue = compatible
```

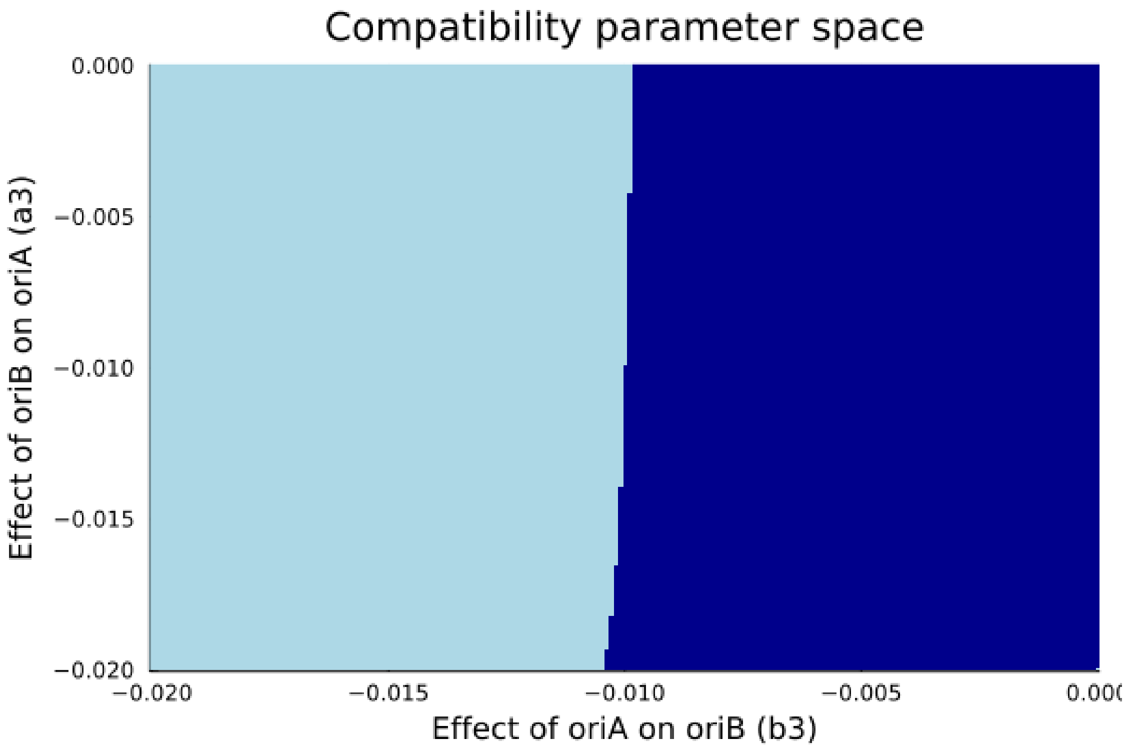

Analysing 2-plasmid populations based on the dPCR data obtained

```
## From our dPCR data we have that:

#=  G4: a2 = 1/48
  F4: a2 = 1/73
  D4.2: a2 = 1/117
  G6: a2 = 1/203
  D4.1: a2 = 1/441
  WT: a2 = 1/774
  when in the presence of antibiotics in the media.

  Let's say that antibiotic selection doubles the copy number of a plasmid
.
  The nature of the change is not important, as long as there is a change.
  =#
```

Example 1A: D4.1 (A) vs G6 (B) - with G6 under selection

```
a2 = 2/441 ## a1/a2 is the carrying capacity of plasmid A
b2 = 1/203 ## b1/b2 is the carrying capacity of plasmid B
a0 = 1/a2
b0 = 1/b2


## Solving the predicted interaction across a large window of interaction
parameters
a3_range = -0.02:0.0001:0  # sets up the range of the investigation
b3_range = -0.02:0.0001:0

results_A = zeros(length(a3_range), length(b3_range)) # prepares a matrix
for entering the results
results_B = zeros(length(a3_range), length(b3_range))
```

```
for i = 1:length(a3_range)
    for j = 1:length(b3_range)
        a3 = a3_range[i]
        b3 = b3_range[j]
        p = [a1, a2, a3, b1, b2, b3]
        u0 = [a0, b0];
        tspan = (0.0, 100.0)  # gives the model 100 units of time to run
        problem = ODEProblem(two_plasmid_competition!, u0, tspan, p)
        solution = solve(problem, AutoVern7(Rodas5()), dt=0.1)

        results_A[i,j] = solution(100)[1]
        results_B[i,j] = solution(100)[2]
    end
end

## Determining areas of co-existence
compatibility_d41tog6 = zeros(length(a3_range), length(b3_range)) # prepar
es a matrix for entering the results

for n = 1: length(results_A)
    if results_A[n] <= 1 || results_B[n] <= 1
        #= Because the system is continuous, it tolerates very small numbe
rs which would have no real meaning
        in a discrete system. As such, we have used here a cut-off of 1, b
elow which the discrete nature of the real system
        would break down =#
        compatibility_d41tog6[n] = 0.0
    else
        compatibility_d41tog6[n] = 1.0
    end
end
heatmap(a3_range, b3_range, compatibility_d41tog6, c = :blues, colorbar=fa
lse, xlabel = "Effect of D4.1 on G6 (b3)",
ylabel = "Effect of G6 on D4.1 (a3)", title = "Compatibility parameter spa
ce")

# light blue = incompatible, dark blue = compatible
```

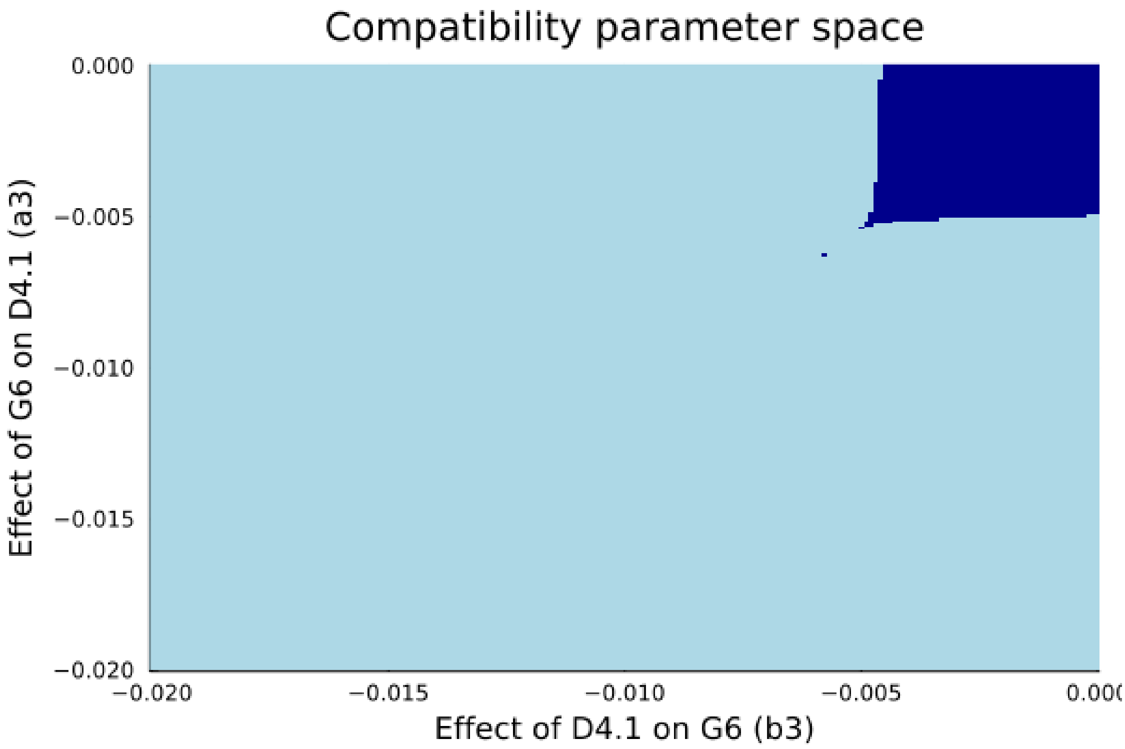

Example 1B: D4.1 (A) vs G6 (B) - with D4.1 under selection

```
a2 = 1/441 ## a1/a2 is the carrying capacity of plasmid A
b2 = 2/203 ## b1/b2 is the carrying capacity of plasmid B
a0 = 1/a2
b0 = 1/b2


## Solving the predicted interaction across a large window of interaction
parameters
a3_range = -0.02:0.0001:0  # sets up the range of the investigation
b3_range = -0.02:0.0001:0

results_A = zeros(length(a3_range), length(b3_range)) # prepares a matrix
for entering the results
results_B = zeros(length(a3_range), length(b3_range))

for i = 1:length(a3_range)
    for j = 1:length(b3_range)
        a3 = a3_range[i]
        b3 = b3_range[j]
        p = [a1, a2, a3, b1, b2, b3]
        u0 = [a0, b0];
        tspan = (0.0, 100.0)  # gives the model 100 units of time to run
        problem = ODEProblem(two_plasmid_competition!, u0, tspan, p)
        solution = solve(problem, AutoVern7(Rodas5()), dt=0.1)

        results_A[i,j] = solution(100)[1]
        results_B[i,j] = solution(100)[2]
    end
end
```

```
## Determining areas of co-existence
compatibility_g6tod41 = zeros(length(a3_range), length(b3_range)) # prepar
es a matrix for entering the results

for n = 1: length(results_A)
    if results_A[n] <= 1 || results_B[n] <= 1
        #= Because the system is continuous, it tolerates very small numbe
rs which would have no real meaning
        in a discrete system. As such, we have used here a cut-off of 1, b
elow which the discrete nature of the real system
        would break down =#
        compatibility_g6tod41[n] = 0.0
    else
        compatibility_g6tod41[n] = 1.0
    end
end
heatmap(a3_range, b3_range, compatibility_g6tod41, c = :blues, colorbar=fa
lse, xlabel = "Effect of D4.1 on G6 (b3)",
ylabel = "Effect of G6 on D4.1 (a3)", title = "Compatibility parameter spa
ce")

# light blue = incompatible, dark blue = compatible
```

```
┌ Warning: Interrupted. Larger maxiters is needed.
└ @ SciMLBase C:\Users\vbbpi\.julia\packages\SciMLBase\Vg9hW\src\integrato
r_interface.jl:331
```

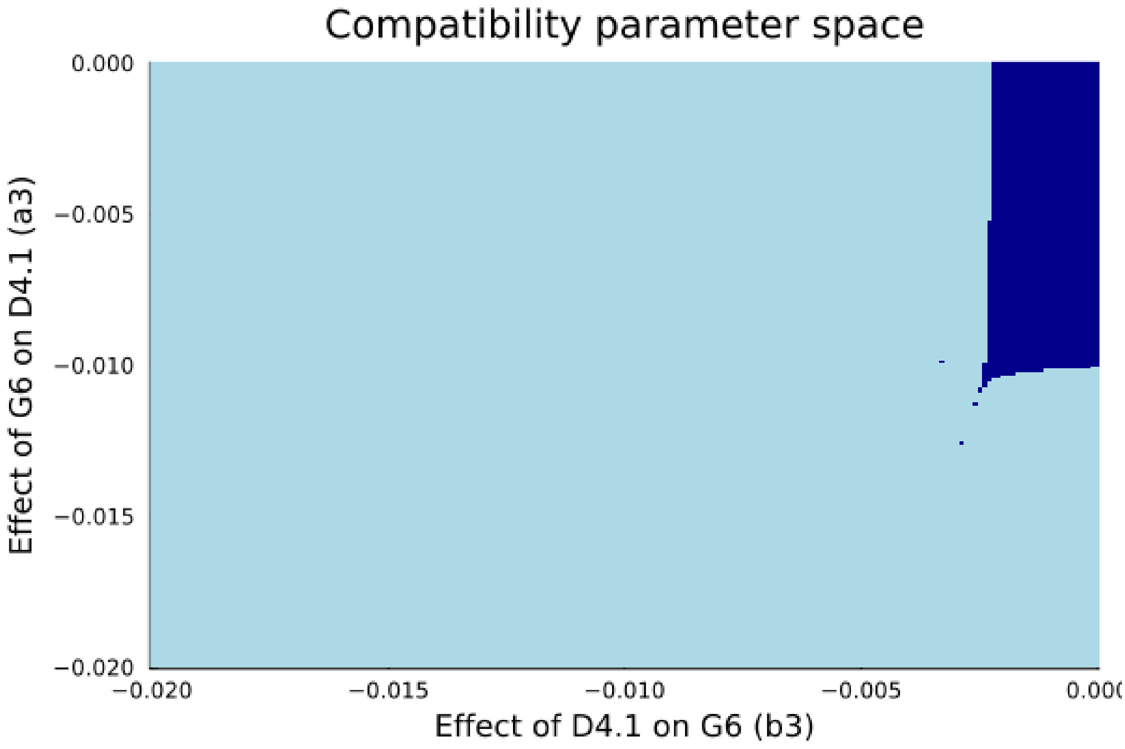

Example 1C: Identifying parameters that satisfy all experimental conditions

```
## Determining area of parameter space that explains the actual data

viable_parameters = zeros(length(a3_range), length(b3_range)) # starting a
```

```
n empty matrix

#= From experimental, we know that when D4.1 is selected, the plasmids are
incompatible.
When G6 is selected, the plasmids are compatible. Therefore the viable par
ameter space
is where compatibility_g6tod41 is 0 but where compatibility_d41tog6 = 1 =#

for n = 1: length(compatibility_d41tog6)
    if compatibility_d41tog6[n] == 1.0 && compatibility_g6tod41[n] == 0.0
        viable_parameters[n] = 1.0
    else
        viable_parameters[n] = 0.0
    end
end

heatmap(a3_range, b3_range, viable_parameters, c = :cividis, colorbar=fals
e, xlabel = "Effect of D4.1 on G6 (b3)",
ylabel = "Effect of G6 on D4.1 (a3)", title = "Experimental parameter spac
e")

# dark blue = not compatible with experimental results; yellow = compatibl
e with experimental results
```

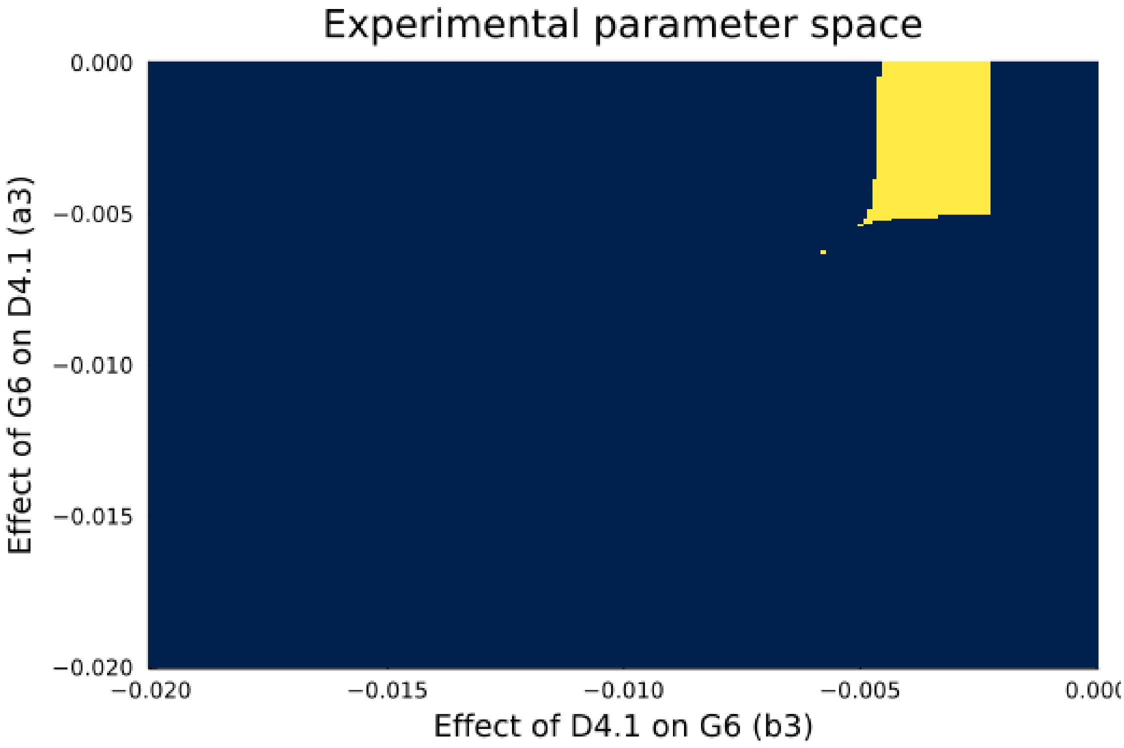

Example 2A: G4 vs WT

This is an important example because it is an intermediate step towards analysing how G4, WT and D4.2 interact.

```
# G4 vs WT - selection on G4
a2 = 1/48 ## a1/a2 is the carrying capacity of plasmid A
b2 = 2/774 ## b1/b2 is the carrying capacity of plasmid B
```

```
a0 = 1/a2
b0 = 1/b2


## Solving the predicted interaction across a large window of interaction
parameters
a3_range = -0.02:0.0001:0  # sets up the range of the investigation
b3_range = -0.02:0.0001:0

results_A = zeros(length(a3_range), length(b3_range)) # prepares a matrix
for entering the results
results_B = zeros(length(a3_range), length(b3_range))

for i = 1:length(a3_range)
    for j = 1:length(b3_range)
        a3 = a3_range[i]
        b3 = b3_range[j]
        p = [a1, a2, a3, b1, b2, b3]
        u0 = [a0, b0];
        tspan = (0.0, 100.0)  # gives the model 100 units of time to run
        problem = ODEProblem(two_plasmid_competition!, u0, tspan, p)
        solution = solve(problem, AutoVern7(Rodas5()), dt=0.1)

        results_A[i,j] = solution(100)[1]
        results_B[i,j] = solution(100)[2]
    end
end

## Determining areas of co-exitence
compatibility_g4towt = zeros(length(a3_range), length(b3_range)) # prepare
s a matrix for entering the results

for n = 1: length(results_A)
    if results_A[n] <= 1 || results_B[n] <= 1
        #= Because the system is continuous, it tolerates very small numbe
rs which would have no real meaning
        in a discrete system. As such, we have used here a cut-off of 1, b
elow which the discrete nature of the real system
        would break down =#
        compatibility_g4towt[n] = 0.0
    else
        compatibility_g4towt[n] = 1.0
    end
end

# G4 vs WT - selection on WT
a2 = 2/48 ## a1/a2 is the carrying capacity of plasmid A
b2 = 1/774 ## b1/b2 is the carrying capacity of plasmid B
a0 = 1/a2
b0 = 1/b2


## Solving the predicted interaction across a large window of interaction
parameters
```

```
a3_range = -0.02:0.0001:0  # sets up the range of the investigation
b3_range = -0.02:0.0001:0

results_A = zeros(length(a3_range), length(b3_range)) # prepares a matrix
for entering the results
results_B = zeros(length(a3_range), length(b3_range))

for i = 1:length(a3_range)
    for j = 1:length(b3_range)
        a3 = a3_range[i]
        b3 = b3_range[j]
        p = [a1, a2, a3, b1, b2, b3]
        u0 = [a0, b0];
        tspan = (0.0, 100.0)  # gives the model 100 units of time to run
        problem = ODEProblem(two_plasmid_competition!, u0, tspan, p)
        solution = solve(problem, AutoVern7(Rodas5()), dt=0.1)

        results_A[i,j] = solution(100)[1]
        results_B[i,j] = solution(100)[2]
    end
end

## Determining areas of co-exitence
compatibility_wttog4 = zeros(length(a3_range), length(b3_range)) # prepare
s a matrix for entering the results

for n = 1: length(results_A)
    if results_A[n] <= 1 || results_B[n] <= 1
        #= Because the system is continuous, it tolerates very small numbe
rs which would have no real meaning
        in a discrete system. As such, we have used here a cut-off of 1, b
elow which the discrete nature of the real system
        would break down =#
        compatibility_wttog4[n] = 0.0
    else
        compatibility_wttog4[n] = 1.0
    end
end

## Determining area of parameter space that explains the actual data

viable_parameters_g4wt = zeros(length(a3_range), length(b3_range)) # start
ing an empty matrix

#= From experimental, we know that irrespective of selection, these plasmi
ds remain compatible.
Therefore the viable parameter space is where compatibility_wttog4 is 1 an
d where compatibility_g4towt = 1 =#

for n = 1: length(compatibility_wttog4)
    if compatibility_wttog4[n] == 1.0 && compatibility_g4towt[n] == 1.0
        viable_parameters_g4wt[n] = 1.0
    else
        viable_parameters_g4wt[n] = 0.0
```

```
    end
end

heatmap(a3_range, b3_range, viable_parameters_g4wt, c = :cividis, colorbar
=false, xlabel = "Effect of G4 on WT (b3)",
ylabel = "Effect of WT on G4 (a3)", title = "Experimental parameter space"
)
# dark blue = not compatible with experimental results; yellow = compatibl
e with experimental results
```

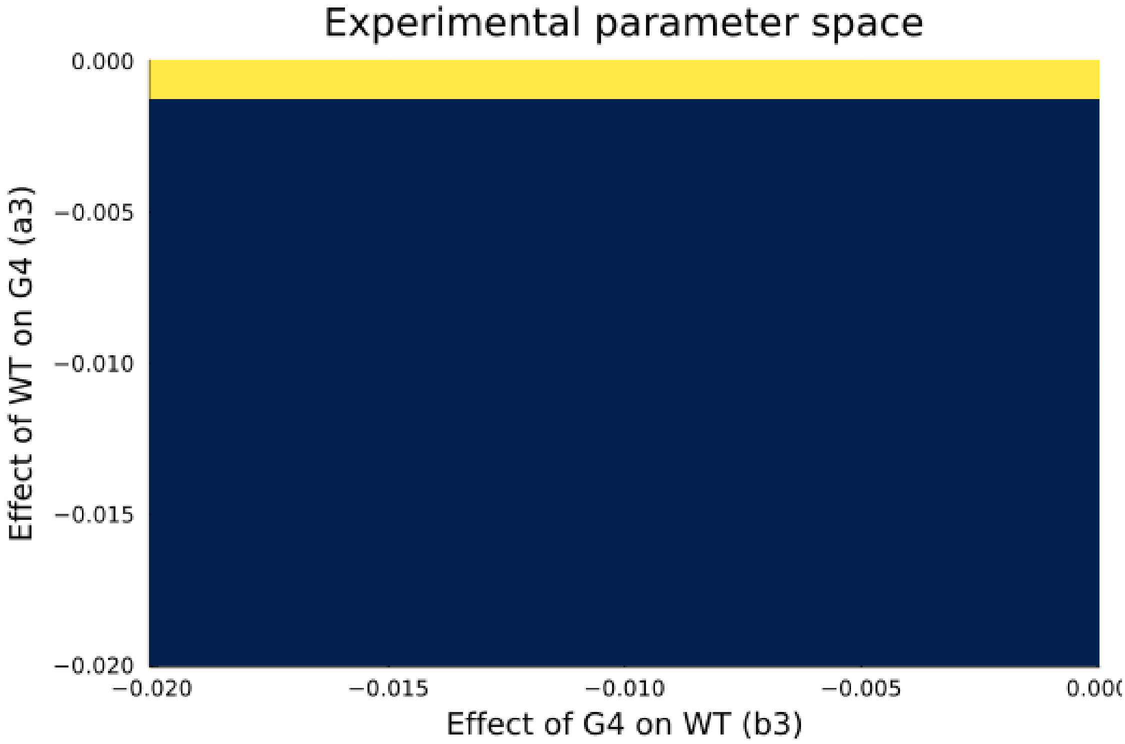

Example 2B: G4 vs. D4.2

```
# G4 vs D4.2 - selection on G4
a2 = 1/48 ## a1/a2 is the carrying capacity of plasmid A
b2 = 2/117 ## b1/b2 is the carrying capacity of plasmid B
a0 = 1/a2
b0 = 1/b2


## Solving the predicted interaction across a large window of interaction
parameters
a3_range = -0.02:0.0001:0  # sets up the range of the investigation
b3_range = -0.02:0.0001:0

results_A = zeros(length(a3_range), length(b3_range)) # prepares a matrix
for entering the results
results_B = zeros(length(a3_range), length(b3_range))

for i = 1:length(a3_range)
    for j = 1:length(b3_range)
        a3 = a3_range[i]
        b3 = b3_range[j]
```

```
        p = [a1, a2, a3, b1, b2, b3]
        u0 = [a0, b0];
        tspan = (0.0, 100.0)  # gives the model 100 units of time to run
        problem = ODEProblem(two_plasmid_competition!, u0, tspan, p)
        solution = solve(problem, AutoVern7(Rodas5()), dt=0.1)

        results_A[i,j] = solution(100)[1]
        results_B[i,j] = solution(100)[2]
    end
end

## Determining areas of co-existence
compatibility_g4tod42 = zeros(length(a3_range), length(b3_range)) # prepar
es a matrix for entering the results

for n = 1: length(results_A)
    if results_A[n] <= 1 || results_B[n] <= 1
        #= Because the system is continuous, it tolerates very small numbe
rs which would have no real meaning
        in a discrete system. As such, we have used here a cut-off of 1, b
elow which the discrete nature of the real system
        would break down =#
        compatibility_g4tod42[n] = 0.0
    else
        compatibility_g4tod42[n] = 1.0
    end
end

# G4 vs D4.2 - selection on D4.2
a2 = 2/48 ## a1/a2 is the carrying capacity of plasmid A
b2 = 1/117 ## b1/b2 is the carrying capacity of plasmid B
a0 = 1/a2
b0 = 1/b2


## Solving the predicted interaction across a large window of interaction
parameters
a3_range = -0.02:0.0001:0  # sets up the range of the investigation
b3_range = -0.02:0.0001:0

results_A = zeros(length(a3_range), length(b3_range)) # prepares a matrix
for entering the results
results_B = zeros(length(a3_range), length(b3_range))

for i = 1:length(a3_range)
    for j = 1:length(b3_range)
        a3 = a3_range[i]
        b3 = b3_range[j]
        p = [a1, a2, a3, b1, b2, b3]
        u0 = [a0, b0];
        tspan = (0.0, 100.0)  # gives the model 100 units of time to run
        problem = ODEProblem(two_plasmid_competition!, u0, tspan, p)
        solution = solve(problem, AutoVern7(Rodas5()), dt=0.1)
```

```
        results_A[i,j] = solution(100)[1]
        results_B[i,j] = solution(100)[2]
    end
end

## Determining areas of co-existence
compatibility_d42tog4 = zeros(length(a3_range), length(b3_range)) # prepar
es a matrix for entering the results

for n = 1: length(results_A)
    if results_A[n] <= 1 || results_B[n] <= 1
        #= Because the system is continuous, it tolerates very small numbe
rs which would have no real meaning
        in a discrete system. As such, we have used here a cut-off of 1, b
elow which the discrete nature of the real system
        would break down =#
        compatibility_d42tog4[n] = 0.0
    else
        compatibility_d42tog4[n] = 1.0
    end
end

## Determining area of parameter space that explains the actual data

viable_parameters_g4d42 = zeros(length(a3_range), length(b3_range)) # star
ting an empty matrix

#= From experimental, we know that irrespective of selection, these plasmi
ds remain compatible.
Therefore the viable parameter space is where compatibility_wttog4 is 1 an
d where compatibility_g4towt = 1 =#

for n = 1: length(compatibility_d42tog4)
    if compatibility_g4tod42[n] == 1.0 && compatibility_d42tog4[n] == 1.0
        viable_parameters_g4d42[n] = 1.0
    else
        viable_parameters_g4d42[n] = 0.0
    end
end

heatmap(a3_range, b3_range, viable_parameters_g4d42, c = :cividis, colorba
r=false, xlabel = "Effect of G4 on D4.2 (b3)",
ylabel = "Effect of D4.2 on G4 (a3)", title = "Experimental parameter spac
e")
# dark blue = not compatible with experimental results; yellow = compatibl
e with experimental results
```

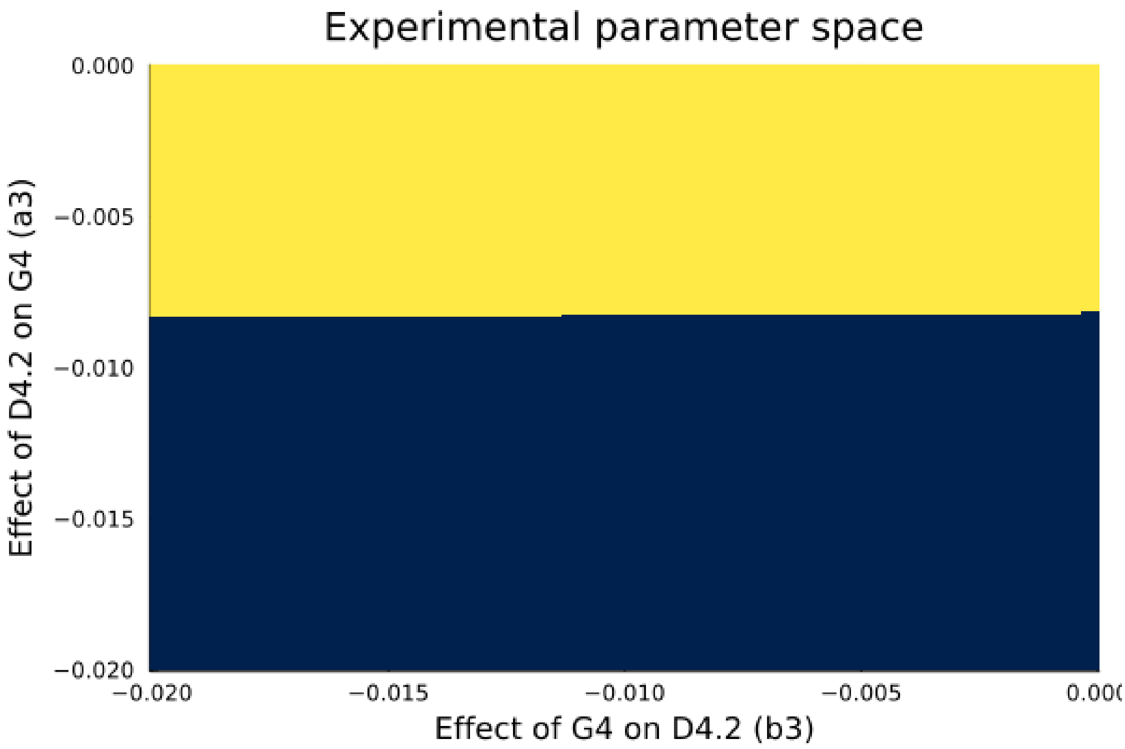

Example 2C: WT vs. D4.2

```
# WT vs D4.2 - selection on WT
a2 = 1/774 ## a1/a2 is the carrying capacity of plasmid A
b2 = 2/117 ## b1/b2 is the carrying capacity of plasmid B
a0 = 1/a2
b0 = 1/b2


## Solving the predicted interaction across a large window of interaction
parameters
a3_range = -0.02:0.0001:0  # sets up the range of the investigation
b3_range = -0.02:0.0001:0

results_A = zeros(length(a3_range), length(b3_range)) # prepares a matrix
for entering the results
results_B = zeros(length(a3_range), length(b3_range))

for i = 1:length(a3_range)
    for j = 1:length(b3_range)
        a3 = a3_range[i]
        b3 = b3_range[j]
        p = [a1, a2, a3, b1, b2, b3]
        u0 = [a0, b0];
        tspan = (0.0, 100.0)  # gives the model 100 units of time to run
        problem = ODEProblem(two_plasmid_competition!, u0, tspan, p)
        solution = solve(problem, AutoVern7(Rodas5()), dt=0.1)

        results_A[i,j] = solution(100)[1]
        results_B[i,j] = solution(100)[2]
    end
end
```

```
## Determining areas of co-existence
compatibility_wttod42 = zeros(length(a3_range), length(b3_range)) # prepar
es a matrix for entering the results

for n = 1: length(results_A)
    if results_A[n] <= 1 || results_B[n] <= 1
        #= Because the system is continuous, it tolerates very small numbe
rs which would have no real meaning
        in a discrete system. As such, we have used here a cut-off of 1, b
elow which the discrete nature of the real system
        would break down =#
        compatibility_wttod42[n] = 0.0
    else
        compatibility_wttod42[n] = 1.0
    end
end

# WT vs D4.2 - selection on D4.2
a2 = 2/774 ## a1/a2 is the carrying capacity of plasmid A
b2 = 1/117 ## b1/b2 is the carrying capacity of plasmid B
a0 = 1/a2
b0 = 1/b2


## Solving the predicted interaction across a large window of interaction
parameters
a3_range = -0.02:0.0001:0  # sets up the range of the investigation
b3_range = -0.02:0.0001:0

results_A = zeros(length(a3_range), length(b3_range)) # prepares a matrix
for entering the results
results_B = zeros(length(a3_range), length(b3_range))

for i = 1:length(a3_range)
    for j = 1:length(b3_range)
        a3 = a3_range[i]
        b3 = b3_range[j]
        p = [a1, a2, a3, b1, b2, b3]
        u0 = [a0, b0];
        tspan = (0.0, 100.0)  # gives the model 100 units of time to run
        problem = ODEProblem(two_plasmid_competition!, u0, tspan, p)
        solution = solve(problem, AutoVern7(Rodas5()), dt=0.1)

        results_A[i,j] = solution(100)[1]
        results_B[i,j] = solution(100)[2]
    end
end

## Determining areas of co-existence
compatibility_d42towt = zeros(length(a3_range), length(b3_range)) # prepar
es a matrix for entering the results

for n = 1: length(results_A)
```

```
    if results_A[n] <= 1 || results_B[n] <= 1
        #= Because the system is continuous, it tolerates very small numbe
rs which would have no real meaning
        in a discrete system. As such, we have used here a cut-off of 1, b
elow which the discrete nature of the real system
        would break down =#
        compatibility_d42towt[n] = 0.0
    else
        compatibility_d42towt[n] = 1.0
    end
end

## Determining area of parameter space that explains the actual data

viable_parameters_wtd42 = zeros(length(a3_range), length(b3_range)) # star
ting an empty matrix

#= From experimental, we know that irrespective of selection, these plasmi
ds remain compatible.
Therefore the viable parameter space is where compatibility_wttog4 is 1 an
d where compatibility_g4towt = 1 =#

for n = 1: length(compatibility_d42tog4)
    if compatibility_d42towt[n] == 1.0 && compatibility_wttod42[n] == 1.0
        viable_parameters_wtd42[n] = 1.0
    else
        viable_parameters_wtd42[n] = 0.0
    end
end

heatmap(a3_range, b3_range, viable_parameters_wtd42, c = :cividis, colorba
r=false, xlabel = "Effect of WT on D4.2 (b3)",
ylabel = "Effect of D4.2 on WT (a3)", title = "Experimental parameter spac
e")
# dark blue = not compatible with experimental results; yellow = compatibl
e with experimental results
```

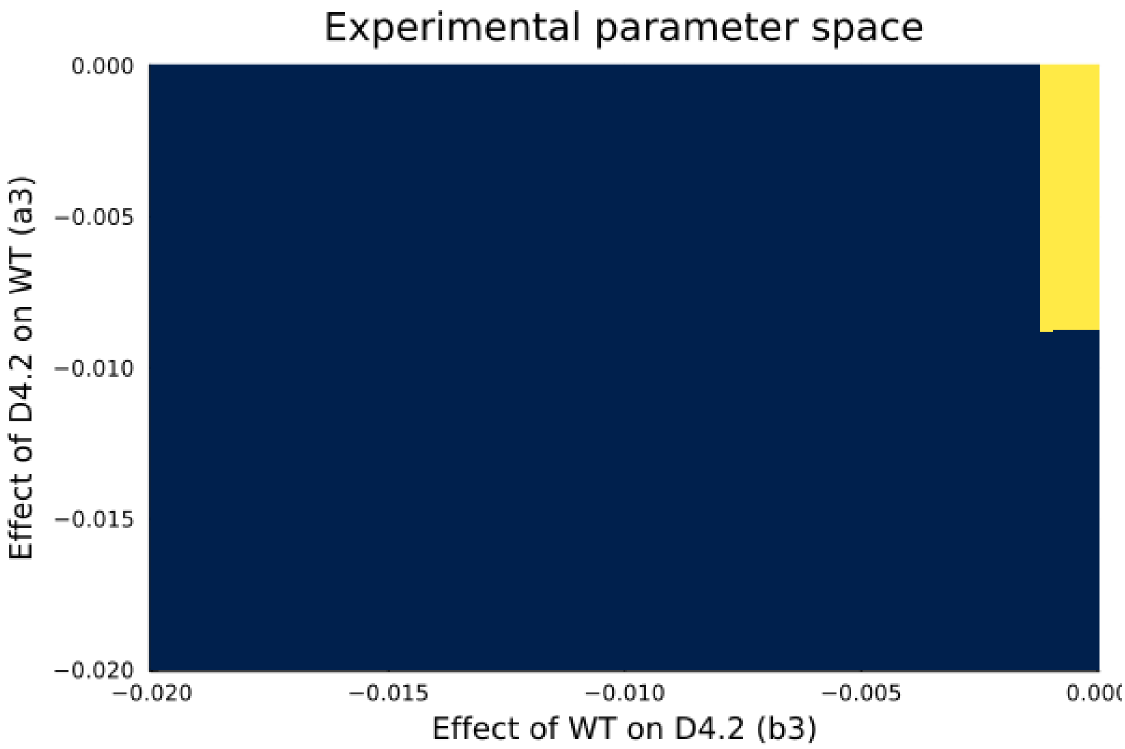

Setting up a 3-population LV system

```
## 3-population LV system
function three_plasmid_competition!(du, u, p, t) ## 2-population generic L
V system
    A, B, C = u
    a1, a2, a3, a4, b1, b2, b3, b5, c1, c2, c4, c5 = p
    du[1] = dA = a1*A - a2*A^2 + a3*A*B + a4*A*C
    #= To improve clarity, indexes were selected to facilitate interaction
identification
    x3 for interactions between A and B, x4 for A and C, and x5 for B and
C =#
    du[2] = dB = b1*B - b2*B^2 + b3*A*B + b5*B*C
    du[3] = dC = c1*C - c2*C^2 + c4*A*C + c5*B*C
end
```

```
three_plasmid_competition! (generic function with 1 method)
```

```
## Parameters
a1 = 1 # for simpliticy chosen as 1
b1 = 1
c1 = 1

a2 = 1/100 # a1/a2 is the carrying capacity of plasmid A
b2 = 1/100 # b1/b2 is the carrying capacity of plasmid B
c2 = 1/100 # c1/c2 is the carrying capacity of plasmid B

a3 = - 1/1000 ## impact of B on A
b3 = - 5/1000 ## impact of A on B
a4 = - 1/1000 ## impact of C on A
c4 = - 4/1000 ## impact of A on C
b5 = - 2/1000 ## impact of C on B
```

```
c5 = - 1/1000 ## impact of B on C

## Initial conditions
a0 = 1/a2
b0 = 1/b2
c0 = 1/c2

## Reformatting parameters for function
p = [a1, a2, a3, a4, b1, b2, b3, b5, c1, c2, c4, c5]
u0 = [a0, b0, c0];

## Solution

#= While the LV system can be solved analytically, we provide here the num
erical solution to avoid having to introduce error
    checking for equilibrium positions that are not in the real positive s
pace for both populations. =#
        tspan = (0.0, 100.0)  # gives the model 100 units of time to run
        problem = ODEProblem(three_plasmid_competition!, u0, tspan, p)
        solution = solve(problem, AutoVern7(Rodas5()), dt=0.1)

        solution(100)  ## returns the two plasmid populations at t = 100
```

```
3-element Vector{Float64}:
 89.70100057995595
 43.18937064960836
 59.80066705330398
```

```
plot(solution, vars=(0,3), linewidth = 3, ylims = (0.0,max(a0,b0)), label
= "C(t)")
plot!(solution, vars=(0,1), linewidth = 3, label = "A(t)")
plot!(solution, vars=(0,2), linewidth = 3, label = "B(t)")
```

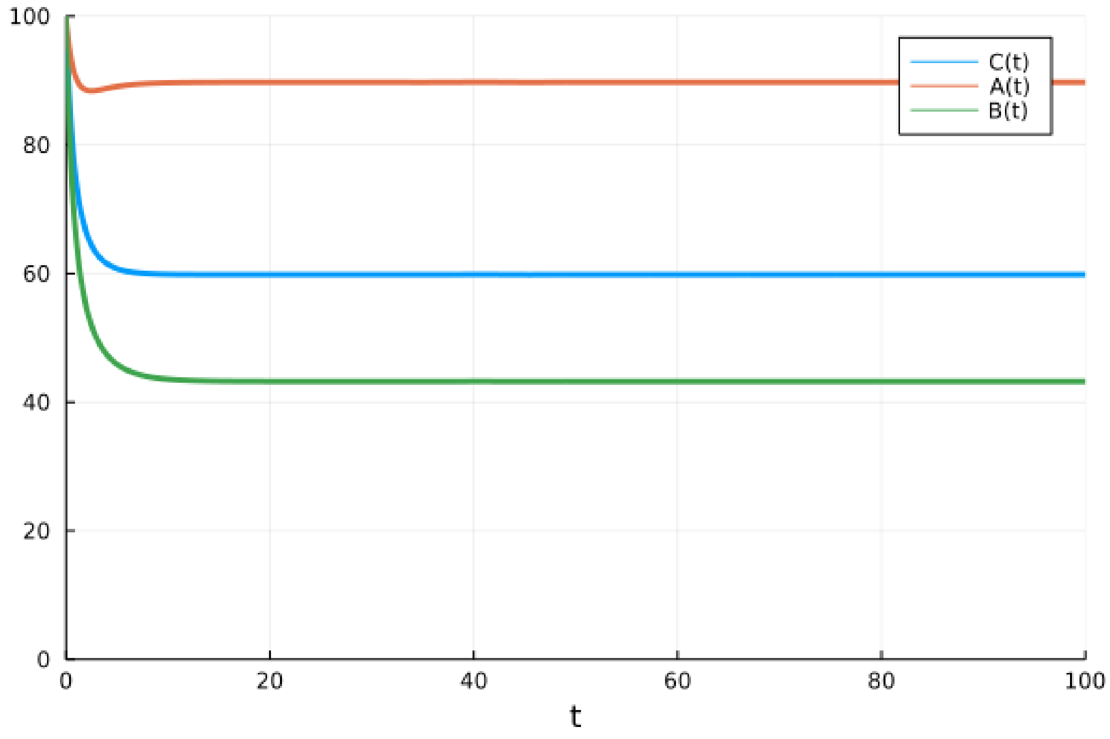

```
## Solving the predicted interaction across a large window of interaction
parameters
#= Unlike the 2-plasmid system with 2 parameters, the 3-plasmid system has
a total of 6 parameters dealing with the interaction
between the origins. That makes it very difficult to explore all at once a
nd to vizualise them all. Below, we show the impact of a
negative impact of plasmid C on plasmid A (i.e. a4) across a wide range of
A and B interactions.=#

a3_range = -0.02:0.0002:0
b3_range = -0.02:0.0002:0
a4_range = -0.02:0.0002:0

results_A = zeros(length(a3_range), length(b3_range), length(a4_range)) #
prepares a tensor for entering the results
results_B = zeros(length(a3_range), length(b3_range), length(a4_range))
results_C = zeros(length(a3_range), length(b3_range), length(a4_range))

for i = 1:length(a3_range)
    for j = 1:length(b3_range)
        for k = 1: length(a4_range)
            a3 = a3_range[i]
            b3 = b3_range[j]
            a4 = a4_range[k]
            p = [a1, a2, a3, a4, b1, b2, b3, b5, c1, c2, c4, c5]
            u0 = [a0, b0, c0];
            tspan = (0.0, 100.0)  # gives the model 100 units of time to r
un
            problem = ODEProblem(three_plasmid_competition!, u0, tspan, p)
            solution = solve(problem, AutoVern7(Rodas5()), dt=0.1)

            results_A[i,j,k] = solution(100)[1]
            results_B[i,j,k] = solution(100)[2]
            results_C[i,j,k] = solution(100)[3]
        end
    end
end

## Determining areas of co-exitence
compatibility_3way = zeros(length(a3_range), length(b3_range), length(a4_r
ange)) # prepares a matrix for entering the results

for n = 1: length(results_A)
    if results_A[n] <= 1 || results_B[n] <= 1 || results_C[n] <= 1
        #= Because the system is continuous, it tolerates very small numbe
rs which would have no real meaning
        in a discrete system. As such, we have used here a cut-off of 1, b
elow which the discrete nature of the real system
        would break down =#
        compatibility_3way[n] = 0.0
    else
        compatibility_3way[n] = 1.0
    end
end
```

```
## Vizualizing the impact of varying parameters on the population
steps = length(a4_range)
t = range(1, length(a4_range), length = steps)

anim = @animate for i ∈ 1:steps
    title_range = "Effect of oriC on oriA, a4=$(a4_range[i])"
    heatmap(a3_range, b3_range, compatibility_3way[i,:,:], c = :thermal, c
olorbar=false, xlabel = "Effect of oriA on oriB (b3)",
    ylabel = "Effect of oriB on oriA (a3)", title = title_range, clims=(0,
1))
end
gif(anim, "compatibility_3way.gif", fps = 5)

# dark blue = not compatible with experimental results; yellow = compatibl
e with experimental results
```

```
┌ Info: Saved animation to
│   fn = c:\Users\vbbpi\OneDrive - KU Leuven\50_Publications\21_New_plasmi
ds\compatibility_3way.gif
└ @ Plots C:\Users\vbbpi\.julia\packages\Plots\1KWPG\src\animation.jl:114

Plots.AnimatedGif("c:\\Users\\vbbpi\\OneDrive - KU Leuven\\50_Publications
\\21_New_plasmids\\compatibility_3way.gif")
```

Using 2-population data to analyse 3-population interactions

```
## Using two-plasmid ranges to look at 3 plasmid interactions
#= Experimental data sets out possible range of parameter values that rela
te to specific interactions between plasmids and therefore
these should remain constant for more complex systems =#

# Using WT(A), D4.2(B) and G4(C), we obtain the following ranges for the p
arameters From the data:
a3_range = -0.01:0.002:0
b3_range = -0.002:0.0005:0
a4_range = -0.02:0.004:0
c4_range = -0.002:0.0005:0
b5_range = -0.02:0.004:0
c5_range = -0.01:0.002:0
```

```
-0.01:0.002:0.0
```

```
compatibility_wt_d42_g4 = zeros(length(a3_range), length(b3_range), length
(a4_range), length(c4_range),
 length(b5_range), length(c5_range));

 #= This creates an empty tensor with dimensions equal to the available ra
nges of parameters being considered.
 Because of the high dimensionality of the data, mapping the individual po
pulations across this paramter landscape
 will not even be attemtped directly.

 Instead, calculations will focus on identifying the presence and size of
viable parameter space for three experimental conditions,
```

```
 while creating a list of possible solutions: =#

possible_param_wt_d42_g4 = Array{Array{Float64 , 1}, 1}(undef,0)

#=
 Example 3: G4, D4.2 and WT being cross-compatible (not the obtained data)
 Example 4: G4, D4.2 and WT leading to loss of G4 (when all populations ar
e under selection)
 Example 5: G4, D4.2 and WT leading to loss of G4 (when only D4.2 is under
selection)
 Example 6: G4, D4.2 and WT leading to loss of G4 (in the absence of selec
tion)
 =#

Vector{Float64}[]
```

Example 3: G4, D4.2 and WT compatible

This is a theoretical example not in agreement with our data (see SI Fig 9C)

```
## Example 3: G4, D42 and WT intercompatible
#= for all three populations to be compatible (while under selection), the
n there must be at least one set of interaction
    parameters for which co-existence is possible, i.e. A(t), B(t) and C(t
) all real and positive.
=#

for a = 1:length(a3_range)
    for b = 1:length(b3_range)
        for i = 1:length(a4_range)
            for j = 1:length(c4_range)
                for x = 1:length(b5_range)
                    for y = 1:length(c5_range)

                        a3 = a3_range[a]
                        b3 = b3_range[b]
                        a4 = a4_range[i]
                        b4 = c4_range[j]
                        b5 = b5_range[x]
                        c5 = c5_range[y]

                        a2 = 1/774
                        b2 = 1/117
                        c2 = 1/48

                        p = p = [a1, a2, a3, a4, b1, b2, b3, b5, c1, c2, c
4, c5]
                        u0 = [a0, b0, c0];
                        tspan = (0.0, 100.0)  # gives the model 100 units
of time to run
                        problem = ODEProblem(three_plasmid_competition!, u
0, tspan, p)
                        solution = solve(problem, AutoVern7(Rodas5()), dt=
0.1)
```

```
                    if solution(100)[1] > 1 && solution(100)[2] > 1 &&
solution(100)[3] > 1
                        compatibility_wt_d42_g4[a,b,i,j,x,y] = 1.0
                        append!(possible_param_wt_d42_g4, [[a3, b3, a4
, c4, b5, c5]])
                    else
                        compatibility_wt_d42_g4[a,b,i,j,x,y] = 0.0
                    end
                end
            end
        end
    end
end
end

solution_found_3way = sum(compatibility_wt_d42_g4)
#= Since each viable solution is assigned the value of 1.0, then the sum g
ives the number of possible combinations tested
for which the conditions are valid =#

2165.0

solution_fraction_3way = solution_found_3way/length(compatibility_wt_d42_g
4)
#= This yields the fraction of the sampled space that is viable
=#

0.06682098765432098

possible_param_wt_d42_g4
#= this returns a list of parameter combinations that fulfil the selection
criteria (here in Example 3 - all plasmids co-existing) =#

2165-element Vector{Vector{Float64}}:
 [-0.01, -0.002, -0.02, -0.004, -0.02, -0.004]
 [-0.01, -0.002, -0.02, -0.004, -0.02, -0.002]
 [-0.01, -0.002, -0.02, -0.004, -0.02, 0.0]
 [-0.01, -0.002, -0.02, -0.004, -0.02, -0.004]
 [-0.01, -0.002, -0.02, -0.004, -0.02, -0.002]
 [-0.01, -0.002, -0.02, -0.004, -0.02, 0.0]
 [-0.01, -0.002, -0.02, -0.004, -0.02, -0.004]
 [-0.01, -0.002, -0.02, -0.004, -0.02, -0.002]
 [-0.01, -0.002, -0.02, -0.004, -0.02, 0.0]
 [-0.01, -0.002, -0.02, -0.004, -0.02, -0.004]
 ⋮
 [-0.004, 0.0, -0.016, -0.004, -0.008, 0.0]
 [-0.004, 0.0, -0.016, -0.004, -0.008, 0.0]
 [-0.004, 0.0, -0.016, -0.004, -0.008, 0.0]
 [-0.004, 0.0, -0.016, -0.004, -0.008, 0.0]
 [-0.002, -0.0005, -0.02, -0.004, -0.008, 0.0]
 [-0.002, -0.0005, -0.02, -0.004, -0.008, 0.0]
 [-0.002, -0.0005, -0.02, -0.004, -0.008, 0.0]
 [-0.002, -0.0005, -0.02, -0.004, -0.008, 0.0]
 [-0.002, -0.0005, -0.02, -0.004, -0.008, 0.0]
```

Example 4: G4, D4.2 and WT leading to loss of G4 (when all populations are under selection)

```
# Example 4: G4, D4.2 and WT leading to loss of G4 (when all populations a
re under selection)
compatibility_wt_d42_nog4 = zeros(length(a3_range), length(b3_range), leng
th(a4_range), length(c4_range),
 length(b5_range), length(c5_range))
possible_param_wt_d42_nog4 = Array{Array{Float64 , 1}, 1}(undef,0)


for a = 1:length(a3_range)
    for b = 1:length(b3_range)
        for i = 1:length(a4_range)
            for j = 1:length(c4_range)
                for x = 1:length(b5_range)
                    for y = 1:length(c5_range)

                        a3 = a3_range[a]
                        b3 = b3_range[b]
                        a4 = a4_range[i]
                        b4 = c4_range[j]
                        b5 = b5_range[x]
                        c5 = c5_range[y]

                        a2 = 1/774
                        b2 = 1/117
                        c2 = 1/48

                        p = p = [a1, a2, a3, a4, b1, b2, b3, b5, c1, c2, c
4, c5]
                        u0 = [a0, b0, c0];
                        tspan = (0.0, 100.0)  # gives the model 100 units
of time to run
                        problem = ODEProblem(three_plasmid_competition!, u
0, tspan, p)
                        solution = solve(problem, AutoVern7(Rodas5()), dt=
0.1)


                        if solution(100)[1] > 1 && solution(100)[2] > 1 &&
solution(100)[3] < 1
                            compatibility_wt_d42_nog4[a,b,i,j,x,y] = 1.0
                            append!(possible_param_wt_d42_nog4, [[a3, b3,
a4, c4, b5, c5]])
                        else
                            compatibility_wt_d42_nog4[a,b,i,j,x,y] = 0.0
                        end
                    end
                end
            end
        end
    end
end
```

```
solution_found_3way_nog4 = sum(compatibility_wt_d42_nog4)
#= Since each viable solution is assigned the value of 1.0, then the sum g
ives the number of possible combinations tested
for which the conditions are valid =#

13725.0

solution_fraction_3way_nog4 = solution_found_3way_nog4/length(compatibilit
y_wt_d42_nog4)
#= This yields the fraction of the sampled space that is viable
=#

0.4236111111111111

possible_param_wt_d42_nog4
#= this returns a list of parameter combinations that fulfil the selection
criteria (here in Example 3 - all plasmids co-existing) =#

13725-element Vector{Vector{Float64}}:
 [-0.01, -0.0015, -0.016, -0.004, -0.02, -0.004]
 [-0.01, -0.0015, -0.016, -0.004, -0.02, -0.004]
 [-0.01, -0.0015, -0.016, -0.004, -0.02, -0.004]
 [-0.01, -0.0015, -0.016, -0.004, -0.02, -0.004]
 [-0.01, -0.0015, -0.016, -0.004, -0.02, -0.004]
 [-0.01, -0.0015, -0.008, -0.004, -0.016, -0.002]
 [-0.01, -0.0015, -0.008, -0.004, -0.016, -0.002]
 [-0.01, -0.0015, -0.008, -0.004, -0.016, -0.002]
 [-0.01, -0.0015, -0.008, -0.004, -0.016, -0.002]
 [-0.01, -0.0015, -0.008, -0.004, -0.016, -0.002]
 ⋮
 [0.0, 0.0, 0.0, -0.004, -0.004, -0.004]
 [0.0, 0.0, 0.0, -0.004, -0.004, -0.002]
 [0.0, 0.0, 0.0, -0.004, -0.004, 0.0]
 [0.0, 0.0, 0.0, -0.004, 0.0, -0.01]
 [0.0, 0.0, 0.0, -0.004, 0.0, -0.008]
 [0.0, 0.0, 0.0, -0.004, 0.0, -0.006]
 [0.0, 0.0, 0.0, -0.004, 0.0, -0.004]
 [0.0, 0.0, 0.0, -0.004, 0.0, -0.002]
 [0.0, 0.0, 0.0, -0.004, 0.0, 0.0]
```

Example 5: G4, D4.2 and WT leading to loss of G4 (when only D4.2 is under selection)

```
# Example 5: G4, D4.2 and WT leading to loss of G4 (when only D4.2 is unde
r selection)
compatibility_wt_d42_nog4_2 = zeros(length(a3_range), length(b3_range), le
ngth(a4_range), length(c4_range),
 length(b5_range), length(c5_range))
possible_param_wt_d42_nog4_2 = Array{Array{Float64 , 1}, 1}(undef,0)


for a = 1:length(a3_range)
    for b = 1:length(b3_range)
        for i = 1:length(a4_range)
            for j = 1:length(c4_range)
                for x = 1:length(b5_range)
                    for y = 1:length(c5_range)
```

```
                        a3 = a3_range[a]
                        b3 = b3_range[b]
                        a4 = a4_range[i]
                        b4 = c4_range[j]
                        b5 = b5_range[x]
                        c5 = c5_range[y]

                        a2 = 2/774
                        b2 = 1/117
                        c2 = 2/48

                        p = p = [a1, a2, a3, a4, b1, b2, b3, b5, c1, c2, c
4, c5]

                        u0 = [a0, b0, c0];
                        tspan = (0.0, 100.0)  # gives the model 100 units
of time to run
                        problem = ODEProblem(three_plasmid_competition!, u
0, tspan, p)
                        solution = solve(problem, AutoVern7(Rodas5()), dt=
0.1)

                        if solution(100)[1] > 1 && solution(100)[2] > 1 &&
solution(100)[3] < 1
                            compatibility_wt_d42_nog4_2[a,b,i,j,x,y] = 1.0
                            append!(possible_param_wt_d42_nog4_2, [[a3, b3
, a4, c4, b5, c5]])
                        else
                            compatibility_wt_d42_nog4_2[a,b,i,j,x,y] = 0.0
                        end
                    end
                end
            end
        end
    end
end


solution_found_3way_nog4_2 = sum(compatibility_wt_d42_nog4_2)
#= Since each viable solution is assigned the value of 1.0, then the sum g
ives the number of possible combinations tested
for which the conditions are valid =#

21300.0

solution_fraction_3way_nog4_2 = solution_found_3way_nog4_2/length(compatib
ility_wt_d42_nog4_2)
#= This yields the fraction of the sampled space that is viable
=#

0.6574074074074074

possible_param_wt_d42_nog4_2
#= this returns a list of parameter combinations that fulfil the selection
criteria (here in Example 3 - all plasmids co-existing) =#
```

```
21300-element Vector{Vector{Float64}}:
 [-0.008, -0.002, -0.02, -0.004, -0.02, -0.01]
 [-0.008, -0.002, -0.02, -0.004, -0.02, -0.008]
 [-0.008, -0.002, -0.02, -0.004, -0.016, -0.01]
 [-0.008, -0.002, -0.02, -0.004, -0.016, -0.008]
 [-0.008, -0.002, -0.02, -0.004, -0.012, -0.01]
 [-0.008, -0.002, -0.02, -0.004, -0.012, -0.008]
 [-0.008, -0.002, -0.02, -0.004, -0.008, -0.01]
 [-0.008, -0.002, -0.02, -0.004, -0.008, -0.008]
 [-0.008, -0.002, -0.02, -0.004, -0.004, -0.01]
 [-0.008, -0.002, -0.02, -0.004, -0.004, -0.008]
 ⋮
 [0.0, 0.0, 0.0, -0.004, -0.004, -0.004]
 [0.0, 0.0, 0.0, -0.004, -0.004, -0.002]
 [0.0, 0.0, 0.0, -0.004, -0.004, 0.0]
 [0.0, 0.0, 0.0, -0.004, 0.0, -0.01]
 [0.0, 0.0, 0.0, -0.004, 0.0, -0.008]
 [0.0, 0.0, 0.0, -0.004, 0.0, -0.006]
 [0.0, 0.0, 0.0, -0.004, 0.0, -0.004]
 [0.0, 0.0, 0.0, -0.004, 0.0, -0.002]
 [0.0, 0.0, 0.0, -0.004, 0.0, 0.0]
```

Example 6: G4, D4.2 and WT leading to loss of G4 (in the absence of selection)

```
# Example 6: G4, D4.2 and WT leading to loss of G4 (in the absence of sele
ction)
compatibility_wt_d42_nog4_noab = zeros(length(a3_range), length(b3_range),
length(a4_range), length(c4_range),
 length(b5_range), length(c5_range))
possible_param_wt_d42_nog4_noab = Array{Array{Float64 , 1}, 1}(undef,0)


for a = 1:length(a3_range)
    for b = 1:length(b3_range)
        for i = 1:length(a4_range)
            for j = 1:length(c4_range)
                for x = 1:length(b5_range)
                    for y = 1:length(c5_range)

                        a3 = a3_range[a]
                        b3 = b3_range[b]
                        a4 = a4_range[i]
                        b4 = c4_range[j]
                        b5 = b5_range[x]
                        c5 = c5_range[y]

                        a2 = 2/774
                        b2 = 2/117
                        c2 = 2/48

                        p = p = [a1, a2, a3, a4, b1, b2, b3, b5, c1, c2, c
4, c5]
                        u0 = [a0, b0, c0];
                        tspan = (0.0, 100.0)  # gives the model 100 units
of time to run
```

```
                        problem = ODEProblem(three_plasmid_competition!, u
0, tspan, p)
                        solution = solve(problem, AutoVern7(Rodas5()), dt=
0.1)

                        if solution(100)[1] > 1 && solution(100)[2] > 1 &&
solution(100)[3] < 1
                            compatibility_wt_d42_nog4_noab[a,b,i,j,x,y] =
1.0
                            append!(possible_param_wt_d42_nog4_noab, [[a3,
b3, a4, c4, b5, c5]])
                        else
                            compatibility_wt_d42_nog4_noab[a,b,i,j,x,y] =
0.0
                        end
                    end
                end
            end
        end
    end
end

solution_found_3way_nog4_noab = sum(compatibility_wt_d42_nog4_noab)
solution_fraction_3way_nog4_noab = solution_found_3way_nog4_noab/length(co
mpatibility_wt_d42_nog4_noab)
possible_param_wt_d42_nog4_noab
```

```
30275-element Vector{Vector{Float64}}:
 [-0.01, -0.002, -0.02, -0.004, -0.02, -0.01]
 [-0.01, -0.002, -0.02, -0.004, -0.02, -0.008]
 [-0.01, -0.002, -0.02, -0.004, -0.02, -0.006]
 [-0.01, -0.002, -0.02, -0.004, -0.02, -0.004]
 [-0.01, -0.002, -0.02, -0.004, -0.02, -0.002]
 [-0.01, -0.002, -0.02, -0.004, -0.02, 0.0]
 [-0.01, -0.002, -0.02, -0.004, -0.016, -0.01]
 [-0.01, -0.002, -0.02, -0.004, -0.016, -0.008]
 [-0.01, -0.002, -0.02, -0.004, -0.016, -0.006]
 [-0.01, -0.002, -0.02, -0.004, -0.016, -0.004]
 ⋮
 [0.0, 0.0, 0.0, -0.004, -0.004, -0.004]
 [0.0, 0.0, 0.0, -0.004, -0.004, -0.002]
 [0.0, 0.0, 0.0, -0.004, -0.004, 0.0]
 [0.0, 0.0, 0.0, -0.004, 0.0, -0.01]
 [0.0, 0.0, 0.0, -0.004, 0.0, -0.008]
 [0.0, 0.0, 0.0, -0.004, 0.0, -0.006]
 [0.0, 0.0, 0.0, -0.004, 0.0, -0.004]
 [0.0, 0.0, 0.0, -0.004, 0.0, -0.002]
 [0.0, 0.0, 0.0, -0.004, 0.0, 0.0]
```

Combining multiple datasets to further refine parameter estimation

```
#= Because we have selections with full antibiotics, with only chloramphen
icol, and without antibiotics, we can intersect the different
sets of parameters to further restrict the range of possible parameters.
```

```
Paramter sets (let it be called p_possible) compatible with all the data g
enerated will be:
    p_possible ∈ possible_param_wt_d42_nog4
    p_possible ∈ possible_param_wt_d42_nog4_2
    p_possible ∈ possible_param_wt_d42_nog4_noab

=#
p_possible = intersect(possible_param_wt_d42_nog4, possible_param_wt_d42_n
og4_noab, possible_param_wt_d42_nog4_2)

2441-element Vector{Vector{Float64}}:
 [-0.008, -0.002, -0.02, -0.004, 0.0, -0.008]
 [-0.008, -0.0015, -0.02, -0.004, -0.008, -0.008]
 [-0.008, -0.0015, -0.02, -0.004, -0.004, -0.008]
 [-0.008, -0.0015, -0.02, -0.004, 0.0, -0.008]
 [-0.008, -0.0015, -0.016, -0.004, 0.0, -0.008]
 [-0.008, -0.001, -0.02, -0.004, -0.02, -0.01]
 [-0.008, -0.001, -0.02, -0.004, -0.02, -0.008]
 [-0.008, -0.001, -0.02, -0.004, -0.016, -0.01]
 [-0.008, -0.001, -0.02, -0.004, -0.016, -0.008]
 [-0.008, -0.001, -0.02, -0.004, -0.012, -0.01]
 ⋮
 [0.0, 0.0, 0.0, -0.004, -0.004, -0.004]
 [0.0, 0.0, 0.0, -0.004, -0.004, -0.002]
 [0.0, 0.0, 0.0, -0.004, -0.004, 0.0]
 [0.0, 0.0, 0.0, -0.004, 0.0, -0.01]
 [0.0, 0.0, 0.0, -0.004, 0.0, -0.008]
 [0.0, 0.0, 0.0, -0.004, 0.0, -0.006]
 [0.0, 0.0, 0.0, -0.004, 0.0, -0.004]
 [0.0, 0.0, 0.0, -0.004, 0.0, -0.002]
 [0.0, 0.0, 0.0, -0.004, 0.0, 0.0]

#= Assuming that the continuous selection of antibiotics must yield cells
with all 3 plasmids (i.e. SI Figure 9C being wrong), then
paramter sets (let it be called p_possible2) compatible with all the data
generated will be:
    p_possible2 ∈ possible_param_wt_d42_g4
    p_possible2 ∈ possible_param_wt_d42_nog4_2
    p_possible2 ∈ possible_param_wt_d42_nog4_noab

resulting in: =#
p_possible = intersect(possible_param_wt_d42_g4, possible_param_wt_d42_nog
4_noab, possible_param_wt_d42_nog4_2)

2-element Vector{Vector{Float64}}:
 [-0.006, -0.001, -0.016, -0.004, -0.004, -0.004]
 [-0.002, -0.0005, -0.02, -0.004, -0.008, 0.0]

#= In conclusion, LV sysmtes are able to fully explain the data providing
testable hypotheses that can be further explored in the lab.
Crucially, it permits interaction between plasmids to be asymmetric (i.e.
orthogonality itself is directional) and quantitative. It is
also likely that robustness of the system can be linked to the permissible
```

parameter space and to how metabolic burdens lead to fluctuation on the number of plasmids per cell. =#
